# Monitub: A Low-Cost Real-Time System for Long-Term Methane Emissions Monitoring from Inland Waterbodies

**DOI:** 10.3390/s26165187

**Published:** 2026-08-16

**Authors:** Brendon Duncan, Alistair Grinham, Matthew D’Souza, Nathaniel Deering

**Affiliations:** 1 School of Civil Engineering, The University of Queensland, St Lucia, Brisbane, QLD 4072, Australia; 2 Fluvio Pty Ltd., Brisbane, QLD 4064, Australia; 3 School of Electrical Engineering and Computer Science, The University of Queensland, St Lucia, Brisbane, QLD 4072, Australia

**Keywords:** methane, long-term monitoring, low-cost sensor, automated floating chamber

## Abstract

Inland waterbodies are a significant emitter of greenhouse gases, especially methane. They contribute 15.2% of all anthropogenic methane emissions. Major uncertainties are still present in emissions estimates for these systems; as a result, more in situ measurements are required to constrain them, especially when ebullition, a direct release of methane, is present. The Monitub Automated Floating Chamber (AFC) was developed to address existing challenges in floating chamber deployments, a common emissions measurement method, through the use of a custom floating chamber, datalogger, and generic calibration of the TGS2611-E00 sensor. This system is able to perform long-term, real-time monitoring of methane emissions from waterbodies at a low cost. This allows for the correlation of environmental drivers to further constrain uncertainties related to emissions, due to its high temporal frequency, and help to answer questions about the spatial heterogeneity of emissions, as more systems can be deployed at a lower cost. The Monitub AFC was deployed to an urban lake for testing, showing its ability to differentiate between diffusion and ebullition, and its capabilities as a long-term monitoring system.

## 1. Introduction

Inland waterbodies are major anthropogenic greenhouse gas (GHG) emission sources [1,2,3]. Methane (${\mathrm{CH}}_{4}$) is the second most emitted GHG, contributing 17.4–19.6% of yearly GHG emissions when converted to carbon dioxide (${\mathrm{CO}}_{2}$) equivalent units [4,5,6]. Inland waterbodies contribute 15.2% of global anthropogenic ${\mathrm{CH}}_{4}$ emissions [7], with 4.3–5.2% emitted from reservoirs [2,7]. Quantifying these emissions is highly important as ${\mathrm{CH}}_{4}$ has a global warming potential 28–34 times higher than ${\mathrm{CO}}_{2}$ over a 100-year time period [8,9]. ${\mathrm{CH}}_{4}$ is produced through methanogenesis from organic carbons in anoxic sediments. This ${\mathrm{CH}}_{4}$ is then emitted into the atmosphere through two major pathways, diffusion and ebullition [10,11]. Diffusion is the process by which produced ${\mathrm{CH}}_{4}$ dissolves into the water column, then slowly diffuses across the water–air boundary. Ebullition occurs when ${\mathrm{CH}}_{4}$ forms small pockets of gas within the sediment matrix which are released when they grow to a sufficient size. After being released, they rise through the water column, representing a direct release of ${\mathrm{CH}}_{4}$ to the atmosphere. Diffusion is present across the entire surface of a reservoir, whilst ebullition is concentrated in areas of high-organic-matter deposits, normally in shallow regions (<5 m) [12,13,14,15]. However, due to the nature of ebullition, the resultant areal emission of ${\mathrm{CH}}_{4}$ is orders of magnitude greater than diffusion [15,16,17], leading to the majority of variability being spatial, both within and between waterbodies [17,18,19]. This is exemplified by one study which showed that 97% of the ${\mathrm{CH}}_{4}$ emissions from a specific reservoir came from only 7% of its surface area due to the presence of ebullition [16]. Large uncertainties surrounding the spatial heterogeneity and temporal dynamics of ${\mathrm{CH}}_{4}$ exist, especially when ebullition is present, with more long-term in situ data required to constrain these uncertainties [7,18,20,21].

In situ measurements of ${\mathrm{CH}}_{4}$ emissions can be taken through different methods, namely, using a floating chamber (FC) [17,22,23,24,25], bubble funnels [26,27,28], or eddy covariance towers [29,30,31,32]. FCs are the simplest method that can capture total emissions (diffusive and ebullition), as well as provide a direct measurement of a known area [33,34]. Using an FC for quantifying ${\mathrm{CH}}_{4}$ emissions is a well-documented method [17,25,35]; however, these methods are labour-intensive, with field personnel required to manually collect samples. Also, direct attribution of emissions to specific pathways is not possible because only a single rate is produced by the method. Automated floating chambers (AFCs) are a subset of FC with integrated sensing technology providing the ability to monitor the headspace at a higher frequency, from sub-daily to sub-minutely [13,35,36,37,38,39], without the need to manually sample, thus allowing for direct pathway attribution. Eddy covariance towers and some AFC designs are expensive to build and operate, as they depend on expensive gas detectors ($10,000–$100,000 USD depending on the device [40]) to quantify gas concentrations. The use of manual FCs has a lower initial cost, but can lead to large ongoing costs from repeat visits to site by field personnel to fully quantify spatial and temporal variability. This necessitates the need for a low-cost method to monitor total ${\mathrm{CH}}_{4}$ emissions without the need for continual involvement of field personnel.

Challenges exist in the long-term monitoring of ${\mathrm{CH}}_{4}$ using an AFC, such as high wind flipping the FC [41,42], humidity inside the headspace sometimes reaching more than 100% relative humidity (RH) [42], posing a condensation risk to sensors, and chamber flotation to accommodate for the extra weight, as traditional FC designs are small and lightweight [22,35]. Sensors used in AFCs can vary from portable spectroscopic analysers [13,35] to low-cost sensors situated in the headspace [36]. The use of low-cost sensors is of particular interest as the reduction in unit costs of the AFC provides the ability to increase coverage on inland waterbodies, thus addressing some of the uncertainty about the spatial heterogeneity of ${\mathrm{CH}}_{4}$ emissions. The TGS2611-E00 [43] is a sensor commonly used in low-cost AFC designs [36,38,39,42]. As high temperatures are required for the metal-oxide sensor (${\mathrm{SnO}}_{2}$) to be sufficiently sensitive, it is bonded to an always-on heating element [44,45]. Similar sensors have been shown to have long-term measurement reliability using appropriate calibration methods [46]. Work has been conducted to produce a calibration for this sensor, with concentrations up to 2000 ppm [47]; however, this is thought to be insufficient if ebullition is present, as it is a direct ${\mathrm{CH}}_{4}$ release to the atmosphere. These calibration equations are also fitted to individual sensors, meaning that large calibration datasets are required to calibrate new sensors [36,48]. To address these challenges, the Monitub AFC was developed with the aim to provide low-cost, real-time, long-term monitoring of ${\mathrm{CH}}_{4}$ emissions, through the use of a custom FC, venting sub-system, datalogger, and generic higher concentration calibration of the TGS2611-E00 sensor.

## 2. Materials and Methods

To provide low-cost, real-time monitoring of ${\mathrm{CH}}_{4}$ emissions, the TGS2611-E00 sensor was selected to measure concentrations within the headspace of a custom FC. To reduce the time and data required to complete calibration for new sensors, a more generic form of the equation was used, with a significant increase in calibration ranges, up to ~10,000 ppm. Due to the high probability of condensation within the headspace, and therefore the probability of sensor corrosion, a custom datalogger was developed. The architecture of the datalogger and sensorboards allows for hot-swapping of malfunctioning sensors, and the quick integration of new sensors. Due to the extra weight imposed by the sensing equipment, the custom FC was designed with extra flotation, extending its long-term capabilities. A venting sub-system was also developed, whose purpose is to keep headspace concentrations within sensor limits and equalise the headspace with the atmosphere.

### 2.1. Datalogger

**Figure 1:**
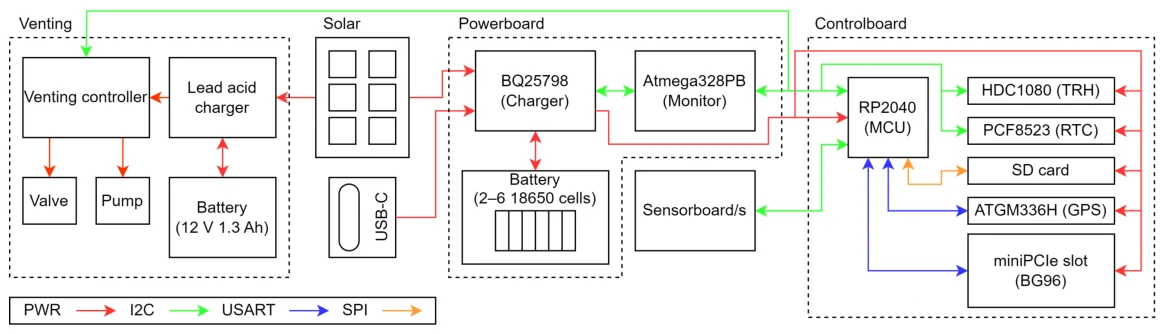
Simplified block diagram of the hardware present in the Monitub datalogger.

The datalogger designed for the Monitub AFC provides all the necessary hardware and software features required for long-term monitoring. The hardware of the datalogger consists of printed circuit boards (PCBs) connecting a central microcontroller, GPS unit, real-time clock (RTC), and SD card to each other and to the sensors (Figure 1). The hardware provides access to the internet through a miniPCIe slot, where a communications card is attached. The main code loop, the software, runs on top of a modified and ported version of MicroPython [49], the firmware. The firmware enables the loading of code from the SD card, providing the ability to add new sensorboards quickly.

#### 2.1.1. Hardware

**Figure 2:**
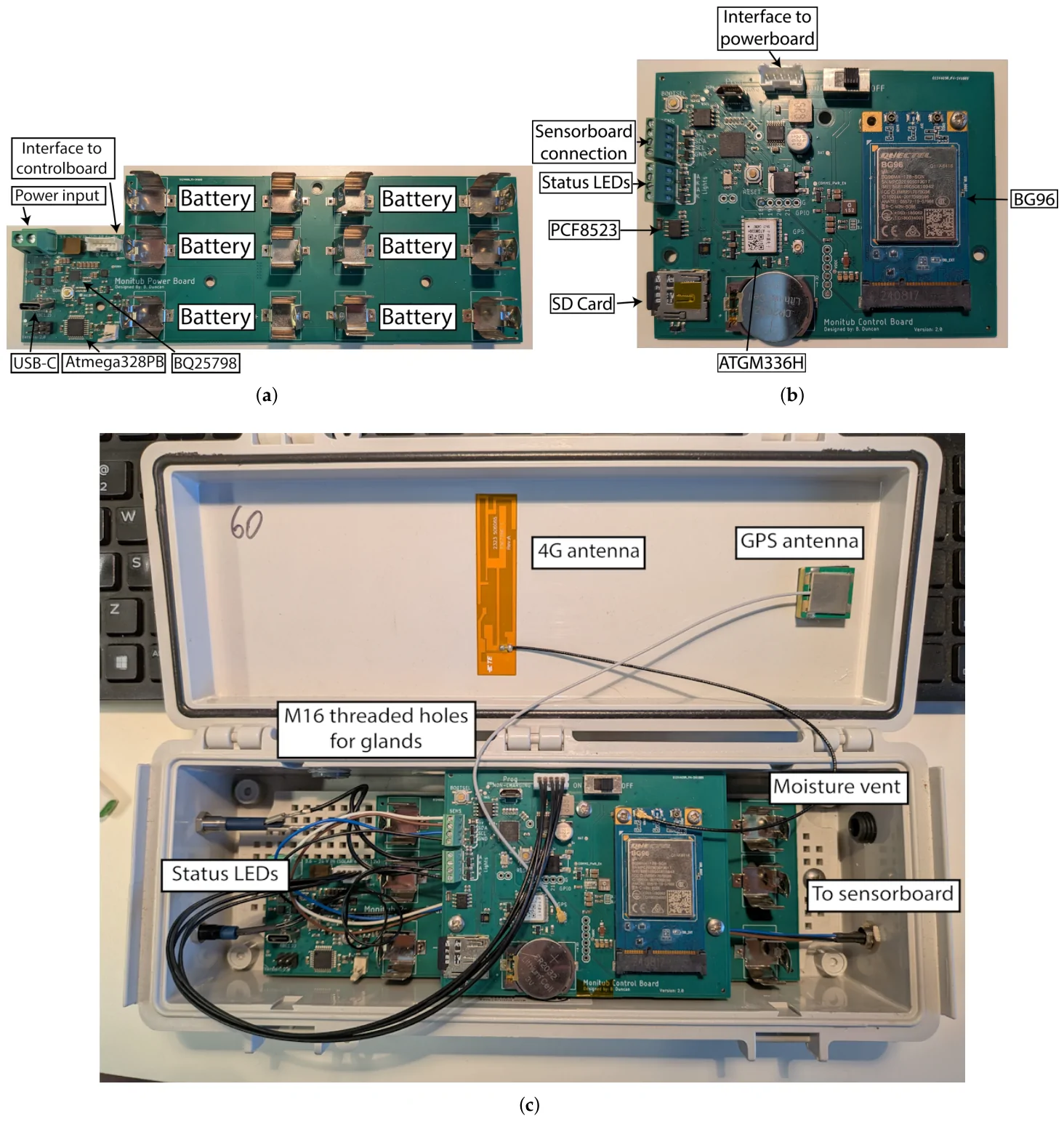
(**a**) The powerboard of the datalogger, with key components labelled. (**b**) The controlboard of the datalogger, with key components labelled. (**c**) The layout of components within the datalogger enclosure.

The hardware of the datalogger is split across 2 PCBs, the controlboard and the powerboard (Figure 2). The separation is required to provide partial replaceability of any datalogger components that may malfunction. The interface between the boards consists of only a power link and an I2C bus connection.

The powerboard is responsible for power management, supplying power to the controlboard, and receiving power from an external source, for example, a solar panel. A charger IC, the BQ25798 [50], was connected to six parallel 18650 Li-Ion batteries. Six batteries were chosen as the selected ${\mathrm{CH}}_{4}$ sensor contains a heater which needs to be constantly powered (0.25 W). The power budget of the datalogger requires an additional 0.25 W, resulting in a running time of up to 115 h (4.8 days) without charging. During deployments, a 20 W solar panel is connected to the powerboard, which charges the batteries in ~3 h. The charger measures all key bus voltages and current, which is monitored by a companion microcontroller, the ATMEGA328PB [51]. The microcontroller uses this to provide an estimation of battery capacity, and to control the power to the controlboard.

The controlboard links all data sources to the core microcontroller, the RP2040 [52], which has two cores, allowing for easier handling of code timeouts. One of the I2C buses of the RP2040 was used to communicate with sensorboards (Section 2.2), as I2C is addressable and allows for multiple sensors to be on the bus without needing additional GPIO lines. The second I2C bus was used to connect to an RTC, the PCF8523 [53], an onboard temperature and humidity (T/RH) sensor, the HDC1080 [54], the powerboard, and the venting sub-system. To provide geolocation and time corrections, a GPS, the ATGM336H [55], was connected to the RP2040 through a USART interface. Finally, a miniPCIe connector with the USART and power pins populated was used to connect the RP2040 to a communication card. Current deployments have used the BG96 CAT-M/NB-IOT miniPCIe card [56]; however, the flexibility of the form factor and the datalogger firmware allows for the quick integration of new cards.

#### 2.1.2. Firmware and Software

**Figure A1:**
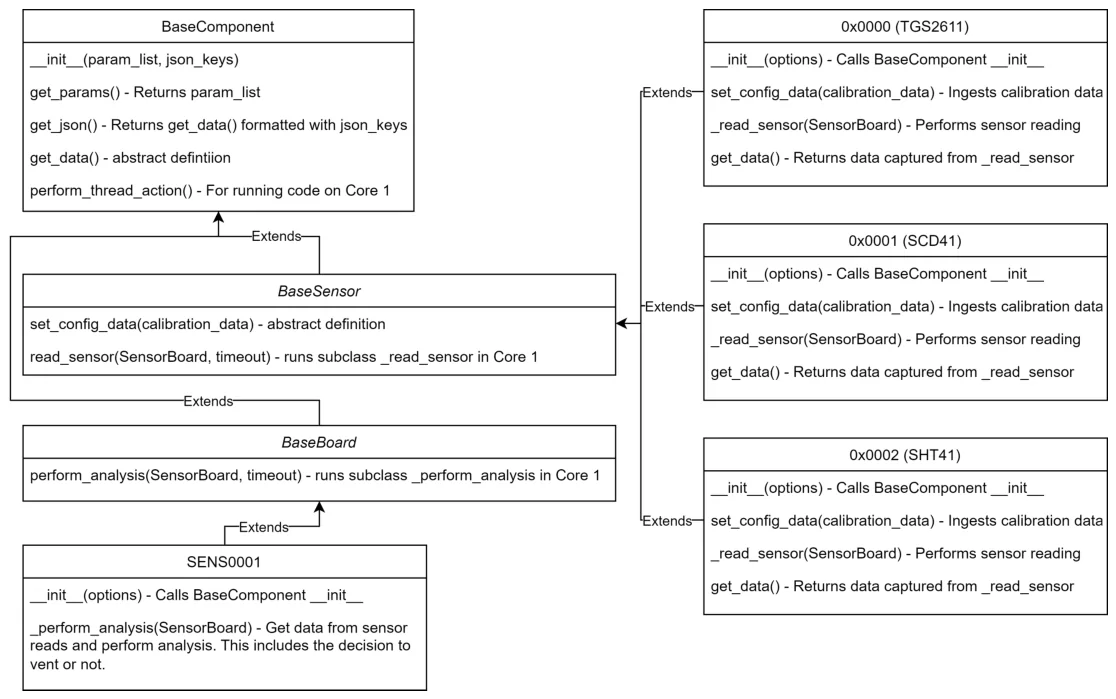
The class structure of sensor codes within the Monitub datalogger software. Providing a common interface for the main datalogger code to interface with new sensors. The example of the sensors used on the SENS-0001 sensorboard is shown.

The initial reason for the selection of the firmware architecture was its ability to load sensor code, a Python code file with a class inheriting from the BaseSensor class (Figure A1), from the SD card. This feature allowed for new sensors to be attached to the datalogger quickly, and with minimal additional lines of code, with an average file length of only ~100 lines. However, the firmware required some modifications from its base port; for the Raspberry Pi Pico, this included modifying the threading component to make use of the second core of the microcontroller for code timeout handling, the addition of a core dump function for debugging purposes, and low-level communications to sensorboard memories (Section 2.2).

**Figure 3:**
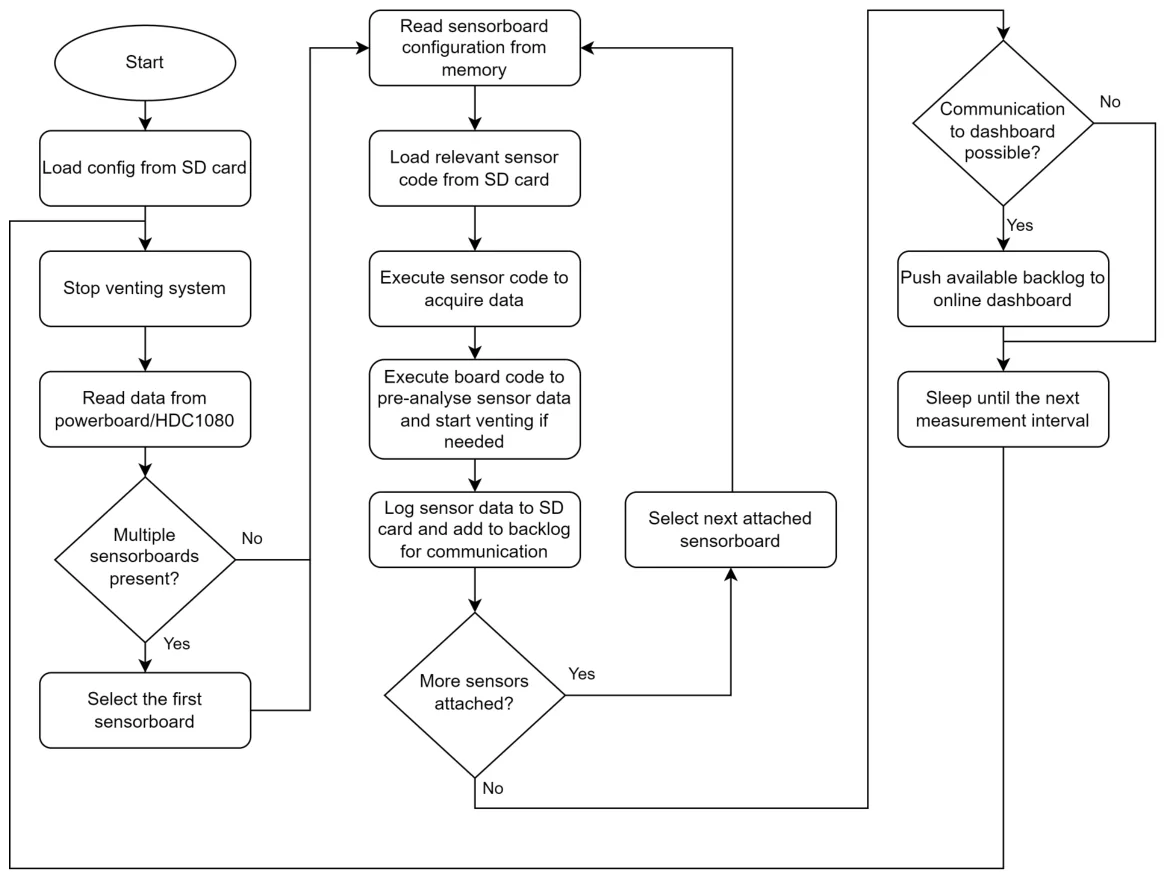
A flow diagram showing an overview of the main logic loop within the software of the Monitub datalogger.

The software running on top of the firmware handles the main code logic, including logging, communications, error handling, and sensor data acquisition (Figure 3). The data acquired from the sensors is stored on the SD card, including all raw values, allowing for later corrections if needed. The software allowed for any object inheriting from a BaseComponent class (parent of BaseSensor class) to be logged, and, thus, pushed to the online dashboard for real-time viewing. The software communicated with the dashboard through the socket and urequests libraries built into the firmware, meaning that the installed communication card is abstracted away from the software.

### 2.2. Sensorboard

A sensorboard consists of any number of environmental sensors, which are connected together to communicate with the Monitub datalogger. Each sensorboard has an associated serial number SENS-tttt-vv-nnnn, where tttt, vv, and nnnn are the type, version and unique number for each sensorboard, respectively. Every sensor on a sensorboard is also given a type, which is not unique to the sensorboard, which is used to connect it to its sampling code on the SD card. Each sensorboard type also has an associated board code; this allows for onboard analysis of the collected sensor data. For this to be fully implemented, a sensorboard must have the following 3 properties:

1. It can be powered by 5 V, with a maximum of 1 A continuous current draw, or have an external power source;
2. It can communicate through I2C (3.3 V TTL);
3. It contains the M24C02 Electrically Erasable Programmable Read-Only Memory (EEPROM) chip, or equivalent.

The EEPROM chip is used to store sensorboard information, sensor types, and each sensors’ specific calibration parameters. This allows for hot-swapping sensorboards in the field without the requirement to reprogram the datalogger. The generic sensorboard memory map can be found in Appendix B.

#### SENS-0001: (${\mathrm{CH}}_{4}$, ${\mathrm{CO}}_{2}$, T/RH)

**Figure 4:**
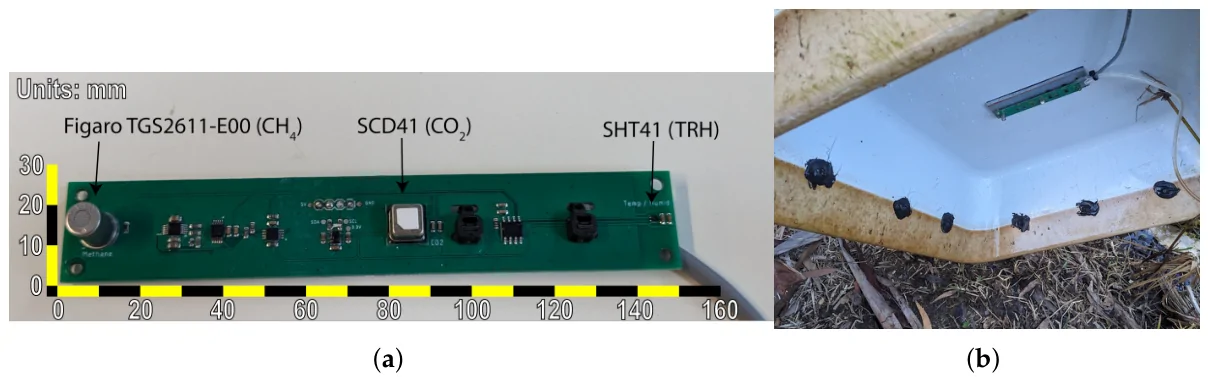
(**a**) The designed ${\mathrm{CH}}_{4}$ sensorboard, with the TGS2611-E00 (${\mathrm{CH}}_{4}$), SCD41 (${\mathrm{CO}}_{2}$) and SHT41 (T/RH) sensors labelled. A scale bar with increments of 10 mm is shown. (**b**) Photo of a sensorboard placed within the headspace of a Monitub AFC.

The SENS-0001 sensorboard was designed to measure ${\mathrm{CH}}_{4}$, ${\mathrm{CO}}_{2}$, temperature, and humidity. The sensors used were the TGS2611-E00 [43], SCD41 [57], and SHT41 [58], respectively (Figure 4a). The resistance of the TGS2611-E00 is related to the concentration of ${\mathrm{CH}}_{4}$. However, the sensor is also sensitive to humidity, and, as such, requires compensation [36,59,60], necessitating the inclusion of the T/RH sensor. The TGS2611-E00 sensor requires additional circuity as it is not directly I2C compatible, made up of a load resistor in series with the sensor placed across the 5 V supply. An analog to digital converter, the ADS1115 [61], measures across the load resistor using a differential channel; this is to limit the error induced by current flowing through the GND plane of the sensorboard, specifically the heater of the TGS2611-E00 which draws ~55 mA [43]. As each SENS-0001 sensorboard was to be deployed within the sealed headspace of an AFC (Figure 4b), which can reach 100%RH due to heating by the sun, an epoxy-based conformal coating was applied to protect it from corrosion.

A calibration equation was required to convert the resistance of the TGS2611-E00 into a gas concentration, whilst also compensating for its sensitivity to humidity. A generic equation approach was chosen to minimise additional parameters required to fit new sensors, also allowing for the calibration to be stored within the sensorboards limited memory. As the Monitub AFC was designed to be used where ebullition is prevalent, a higher calibration range of 2 ppm to 10,000 ppm was used. It was found that Equation (8) from [36] was adequate to compensate for humidity, even with the extended calibration range.

(1) $$Q=aR^{b}\left(1+cH\right)+K$$

where *Q* is the ${\mathrm{CH}}_{4}$ concentration measured by the sensor (ppm), *H* is the absolute humidity (kg $m^{-3}$), *R* is the scaled resistance of the sensor, and *a*, *b*, *c* and *K* are constants. *H* is determined by converting the relative humidity and temperature reported by the SHT41 to absolute humidity (Appendix E.1). The values of *a*, *b*, *c* and *K* were kept the same for all sensors as it was observed that sensitivity to humidity changes were consistent between sensors. The individual sensor calibration was therefore only a matter of compensating for individual sensor concentration dependence. This was achieved through scaling of *R*, specifically

(2) $$R=\frac{R_{s}}{R_{0}}$$

(3) $$R_{s}=R_{L}\left(\frac{V_{cc}}{V_{s}}-1\right)$$

where $R_{s}$ is the sensor resistance (Ω), translated from $V_{s}$ (V), the measured voltage across the load resistor $R_{L}$ (Ω), and $R_{0}$ (Ω) is a constant. This $R_{0}$ constant serves as the calibration difference between sensors. To obtain generic values for *a*, *b*, *c* and *K*, an initial guess of the value of $R_{0}$ for each of the sensors was required; this was set to be the same as the load resistance (i.e., $R_{0}=R_{L}$), which was the recommended value from the manufacturer.

**Figure 5:**
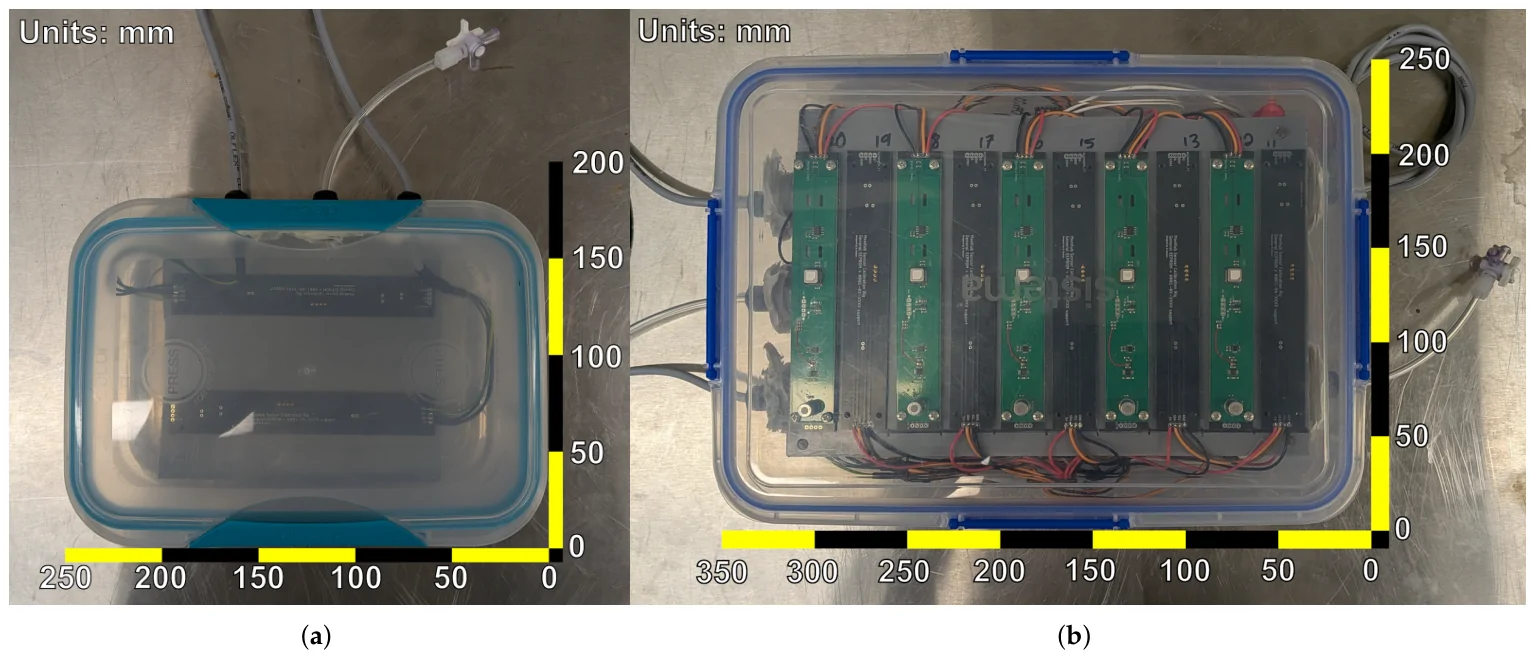
(**a**) A small container (~2 L) mainly used for the initial characterisation of two SENS-0001 sensorboards, ports consist of two communication cables for the sensorboards, and a single tube with stopcock for injection and sampling of gas. (**b**) A larger container (~10 L) used for mass calibration with space to fit twenty SENS-0001 sensorboards sensors, connected to three TCA9548A I2C multiplexer breakout boards. Five ports are present: three for power and communications, and two for tubes with stopcocks for injection and sampling of gas. Scale bars are present for both, with 50 mm increments.

**Figure A3:**
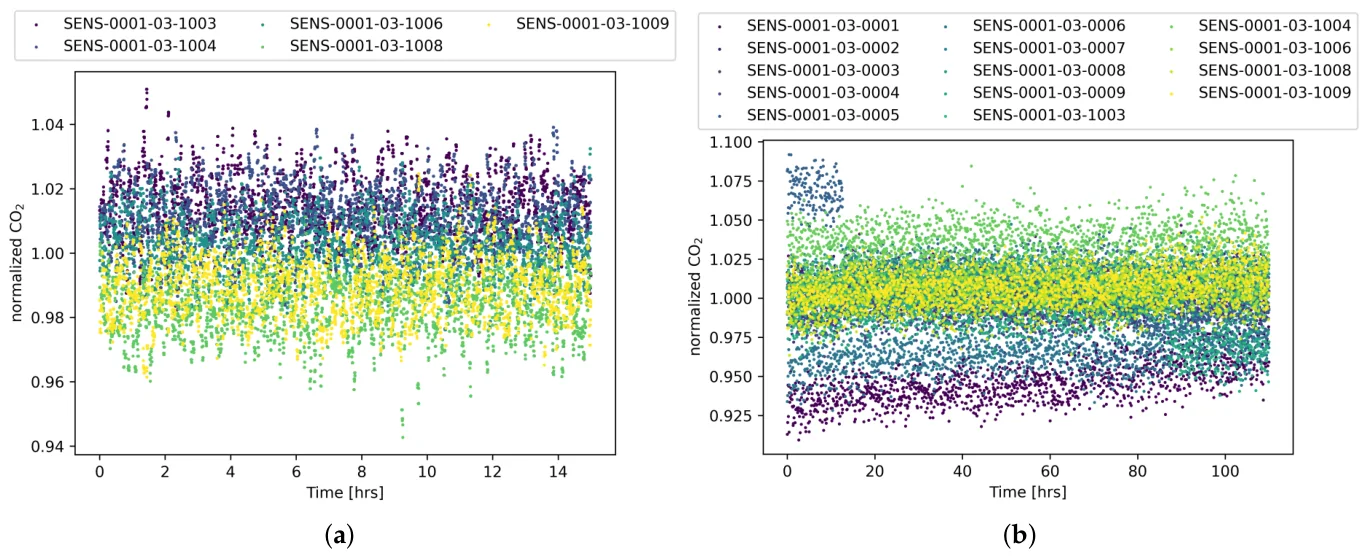
CO_2_ traces captured by the sensors during 2 of the calibration drawdown experiments, (**a**) a 15 h experiment with 5 sensors, and (**b**) a 110 h experiment with 14 sensors. The CO_2_ values have been divided by an average ${\mathrm{CO}}_{2}$ trace created from all sensors during each experiment. There is minimal change in proportions between traces, showing that the headspace of the container is well mixed throughout the duration of the drawdown experiments. Due to the length of experiment in (**b**), the points have been subsampled 100 times for clarity.

The data for the calibration was collected using 28 distinct TGS2611-E00 sensors through 3 different methods. The first method consisted of laboratory experiments, where different volumes of a gas standard were injected into a plastic container containing SENS-0001 sensorboards (Figure 5a). The container was exposed to different temperatures through the use of an oven, and different humidities through the inclusion of a dish of water, or a desiccant packet. Samples of the gas within the container were taken to be later analysed through gas chromatography. These samples were then directly attributed to corresponding electronic samples. The second method consisted of field experiments with a Monitub AFC with a SENS-0001 sensorboard in its headspace, with further gas samples taken to be later analysed and matched to electronic samples. The third data acquisition method was through multiple drawdown experiments, consisting of sealing a number of SENS-0001 sensorboards into a larger plastic container, then injecting an initial volume of gas standard into it, usually increasing concentrations of ${\mathrm{CH}}_{4}$ to 10,000 ppm (Figure 5b). The sensors were logged at 1 Hz, then a stopcock was opened to allow the gas to slowly leak out of the container. A gas sample was periodically taken to provide a curve of the concentrations within the container over the length of the experiments. The container was assumed to be well mixed, with each TGS2611-E00 measuring the same concentration of ${\mathrm{CH}}_{4}$. This was verified by investigating the ${\mathrm{CO}}_{2}$ traces during the drawdown experiments (Figure A3), with the assumption that ${\mathrm{CH}}_{4}$ would mix similarly to ${\mathrm{CO}}_{2}$.

### 2.3. Venting Sub-System

**Figure 6:**
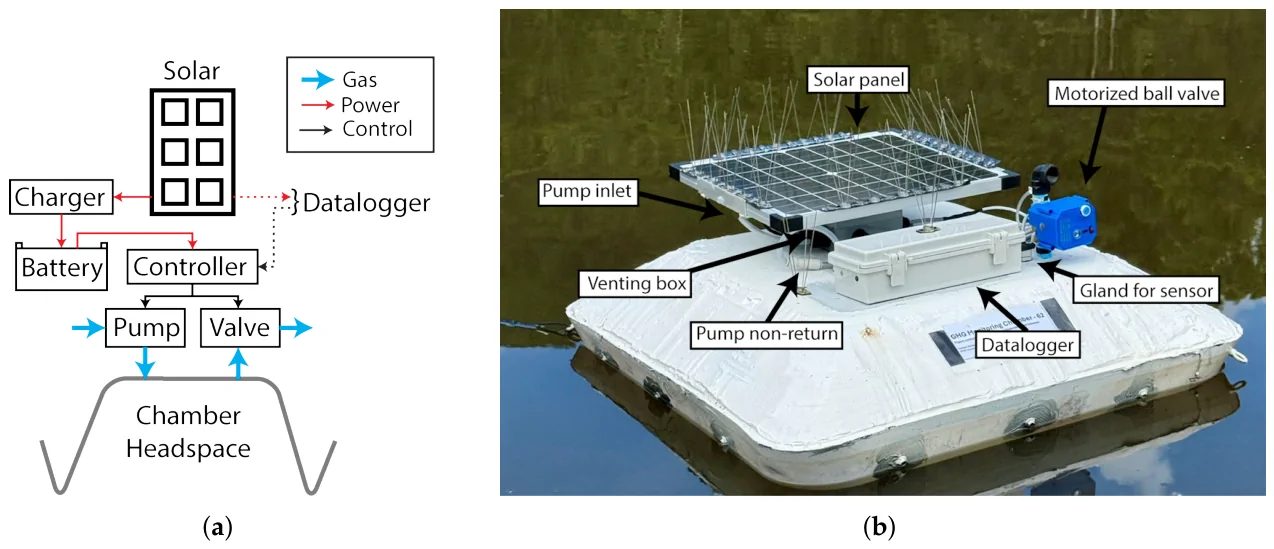
(**a**) Conceptual diagram of the designed venting sub-system, with gas (blue), power (red), and control (black) connections between the components shown with direction. Dashed lines represent interaction with the datalogger. (**b**) Photo of venting sub-system installed on a deployed FC, with labels showing the location of components.

A headspace venting sub-system was required to equalise the headspace of the AFC with the atmosphere. This reduces skewing of measured emission rates as the headspace concentration equalises with the water column beneath the chamber. It also keeps concentrations below the lower explosive limit for safety, and within sensor calibration limits. The venting sub-system has a power source independent of the datalogger, ensuring the risk of killing the datalogger due to excess power usage or part failure is minimised. The system consists of 4 major components (Figure 6):

- An air pump: to pump air into the headspace of the chamber;
- A motorised ball valve: to provide an exit path for replaced gases;
- An independent battery: to supply power to the system;
- Control and charging circuitry: to control the system.

The venting sub-system is controlled through I2C, with the datalogger determining whether a venting operation is required. This occurs after a sample has been taken, with the venting sub-system running till the next sample is ready to be taken, usually after 10 min. Two main venting conditions were used, time and concentration, with exact parameters for each depending on the specific deployment. The time condition forced venting to occur during predefined time periods, whilst the concentration condition activated venting once headspace concentrations surpassed a configured threshold value. A 12 V vacuum pump, available from Sparkfun [62], with a free flow rate of 12–15 L $\min^{-1}$ at a power draw of 12 W, was used to pump air through an 8 mm tube into the headspace. The ball valve was acquired from DFRobot [63] and draws an additional 2 W when actuating. A 1.3 Ah 12 V lead-acid battery was used to power the sub-system, charged through a dedicated charger using the same solar panel as the datalogger. A fully charged battery results in approximately 1 h, or 6 sampling periods of 10 min, of continual venting.

### 2.4. Floating Chamber

**Figure 7:**
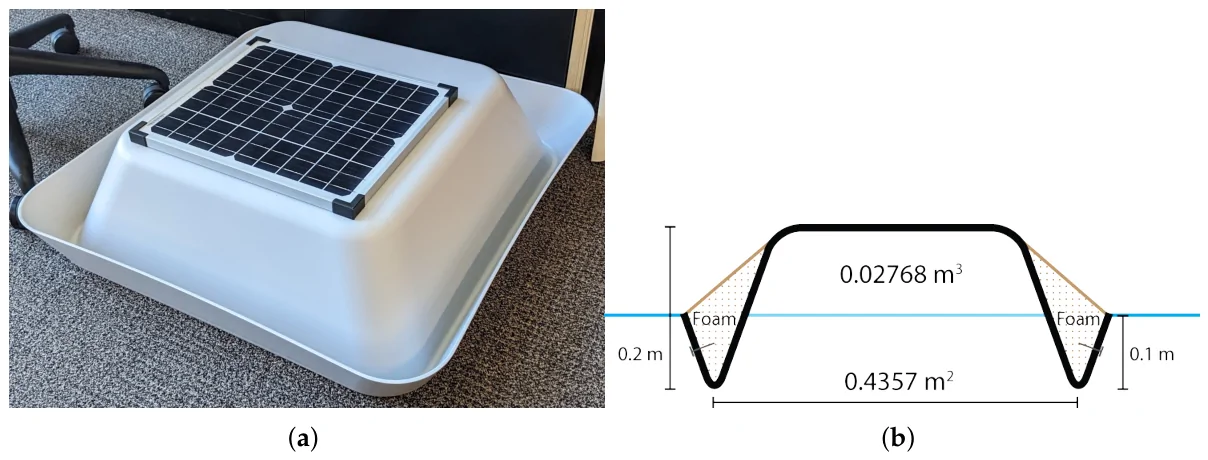
(**a**) Photo of the vacuum formed chamber before foam filling with a 20 W solar panel placed on the payload area. (**b**) Cross-section of the chamber showing the foam fill areas and approximate height in the water (blue line).

Floating chambers were custom designed for mass manufacturability at a relatively low cost (<$150 AUD). A larger payload area atop the chamber was designed for installation of the datalogger, venting sub-system, and solar panel (Figure 7a). Buoyancy is provided by an external cavity which is filled with expanding polyurethane foam, for a total buoyancy of 25 kg when the headspace is unsealed (Figure 7b). The foam is mechanically fixed to the chamber, then sealed with a waterproof liner to avoid water seepage into the foam structure. The total weight of the Monitub AFC is 15 kg, resulting in a measurement area of 0.4357 $m^{2}$, with an internal headspace volume of 0.02768 $m^{3}$ when equalised, and a corresponding area to volume ratio of 16.

### 2.5. Dashboard

**Figure 8:**
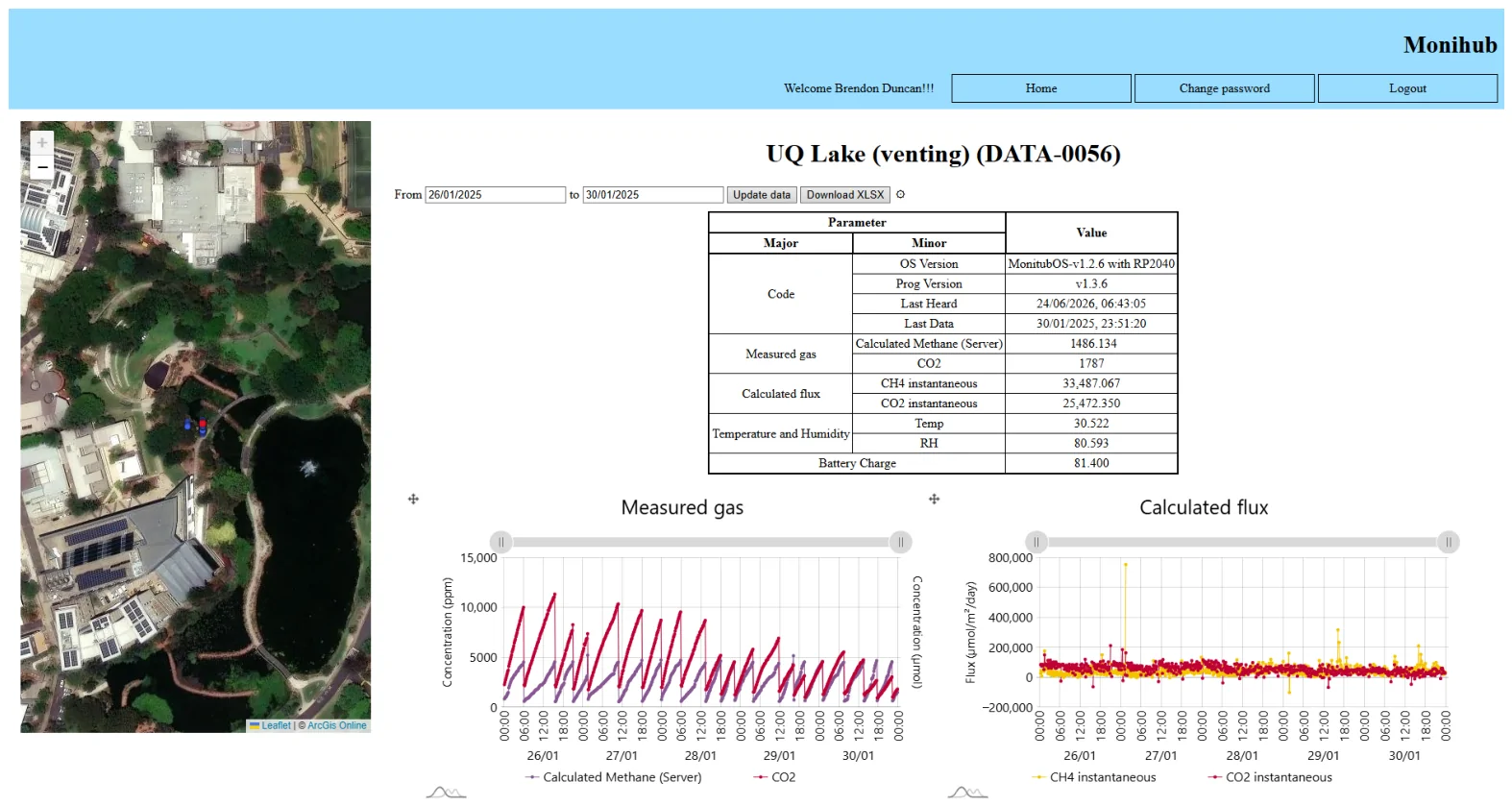
An example dashboard on Monihub showing the data from 26 January 2025 to 30 January 2025 for a system deployed to a lake. Latest values are presented in the table in the centre, with graphs of data following. Two graphs are visible, being the headspace concentrations of ${\mathrm{CH}}_{4}$ and ${\mathrm{CO}}_{2}$ (left), and instantaneous flux estimates (right). Along with this, the reported location of each data point is plotted onto a map (far left), with blue dots representing previous locations, and red dots representing the latest location reported.

Monihub is the dashboard developed for use with the Monitub AFC. It is a custom lightweight dashboard hosted on a server provided by The University of Queensland. It consists of an Nginx server which forwards requests to a Python Flask application. This Flask app handles reception, storage, and display of data, and the sending of configuration updates to Monitub AFCs. Each datalogger has a corresponding dashboard displaying real-time data and post-processed results performed on the server (Figure 8). The post-processed data includes unit conversions (Appendix E.2) and instantaneous emission rate calculations (Appendix E.3). The dashboard can also source location-specific data, such as atmospheric pressure and wind speed, for direct comparison to emissions figures. These atmospheric parameters are also used in the post-processed results to provide localised corrections, such as using local atmospheric pressure to correct unit conversions.

## 3. Results

Results from the generic calibration of the TGS2611-E00 showed a good fit, demonstrating how a more generic calibration equation can still provide accurate results. Deployments of the Monitub AFC were used to validate the calibration of the TGS2611-E00 sensor, with errors from this providing the baseline for uncertainty calculations related to unit conversions (Appendix E.2). The venting sub-system was verified to show that it could adequately control headspace concentrations, to keep within sensor calibrations, and to reduce errors due to equalisation with ${\mathrm{CH}}_{4}$ concentrations in the water column. This all culminated in the long-term deployment of a Monitub AFC to an urban lake at The University of Queensland, showing that it can be used for long-term, real-time ${\mathrm{CH}}_{4}$ monitoring.

### 3.1. Calibration of TGS2611-E00 Sensor

**Figure 9:**
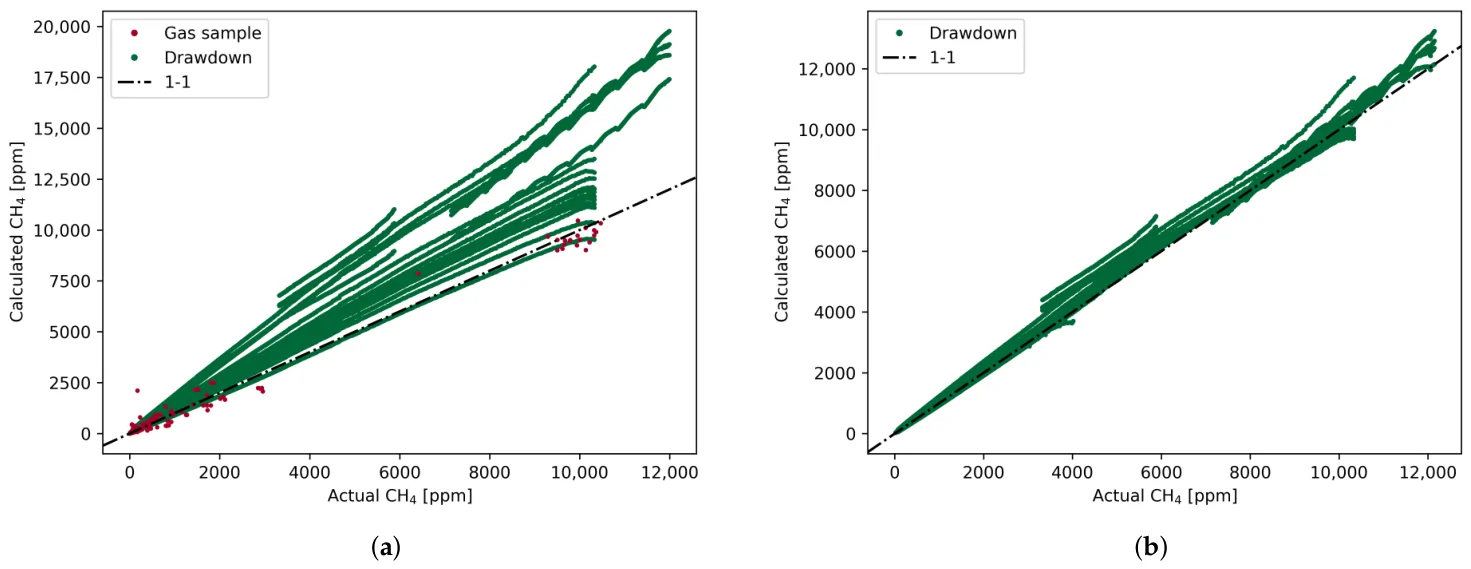
(**a**) The initial data from the gas samples and drawdown experiments shown fit to Equation (1). This fit has $R^{2}=0.512$ and RMSE=2320 ppm. (**b**) Fit for the drawdown experiments when fitting $R_{0}$ for each distinct sensor, resulting in $R^{2}=0.990$ and RMSE=330 ppm.

A total of 225 gas samples were captured from the calibration experiments, with 112 samples from laboratory experiments and 113 from field deployments. Temperatures of these samples ranged from 15.2 °C to 56.9 °C, and relative humidity ranged from 19.9% to 105.0%, as reported by the SHT41. The time series collected during the drawdown experiments was downscaled to every 100th sample, due to the length of each experiment (ranging from 2 to 5 days), resulting in 26,928 additional data points. These experiments experienced a temperature range of 24.5 °C to 42.5 °C and relative humidity ranging from 31.3% to 80.8%. Overall the sensors used in the calibration experienced absolute humidity values ranging from 3.8 g $m^{-3}$ to 57.7 g $m^{-3}$. The data from the sensors was then fit to Equation (1) using curve_fit from the SciPy Python package, and forcing $R_{0}=R_{L}$ for each distinct sensor. The sigma parameter of curve_fit was used to weight the fit, with dedicated gas samples assigned a sigma value of 1, whilst the drawdown experiments were assigned a value of 100. This higher value was selected as the drawdown experiments only used an approximation of the gas concentration, and as they produced significantly more data. This weighting meant that the dedicated samples still influenced the fit, as they experienced more variation in humidity. As a result, the fit was able to smooth out these variations and achieve $R^{2}=0.512$ and root mean square error (RMSE) of 2320 ppm (Figure 9a). The large errors are due to the original assumption of $R_{0}=R_{L}$, with full compensation of each sensors’ sensitivity to ${\mathrm{CH}}_{4}$ not accounted for. This fit provides the generic equation parameters (*a*, *b*, *c* and *K*) which are used for all further sensors, allowing for a single drawdown experiment, or a number of distinct data points, to be used to calibrate a new SENS-0001 sensorboard. The fitted values of the generic equation parameters were

(4) $$\begin{array}{cc} a & =3.75931242\times 10^{3}; \end{array}$$

(5) $$\begin{array}{cc} b & =-2.10543917; \end{array}$$

(6) $$\begin{array}{cc} c & =-9.47074592; \end{array}$$

(7) $$\begin{array}{cc} K & =-1.27398093\times 10^{1}. \end{array}$$

The individual sensor calibration is completed by using curve_fit to fit $R_{0}$ in Equation (2). This further constrains the previous fit, resulting in $R^{2}=0.990$ and RMSE=330 ppm when looking at the drawdown curves (Figure 9b). This shows that a new arbitrary sensor can be calibrated easily, as this would only require the fitting of one parameter ($R_{0}$), and, thus, require a small amount of calibration data.

### 3.2. Validation of TGS2611-E00 Calibration

**Figure 10:**
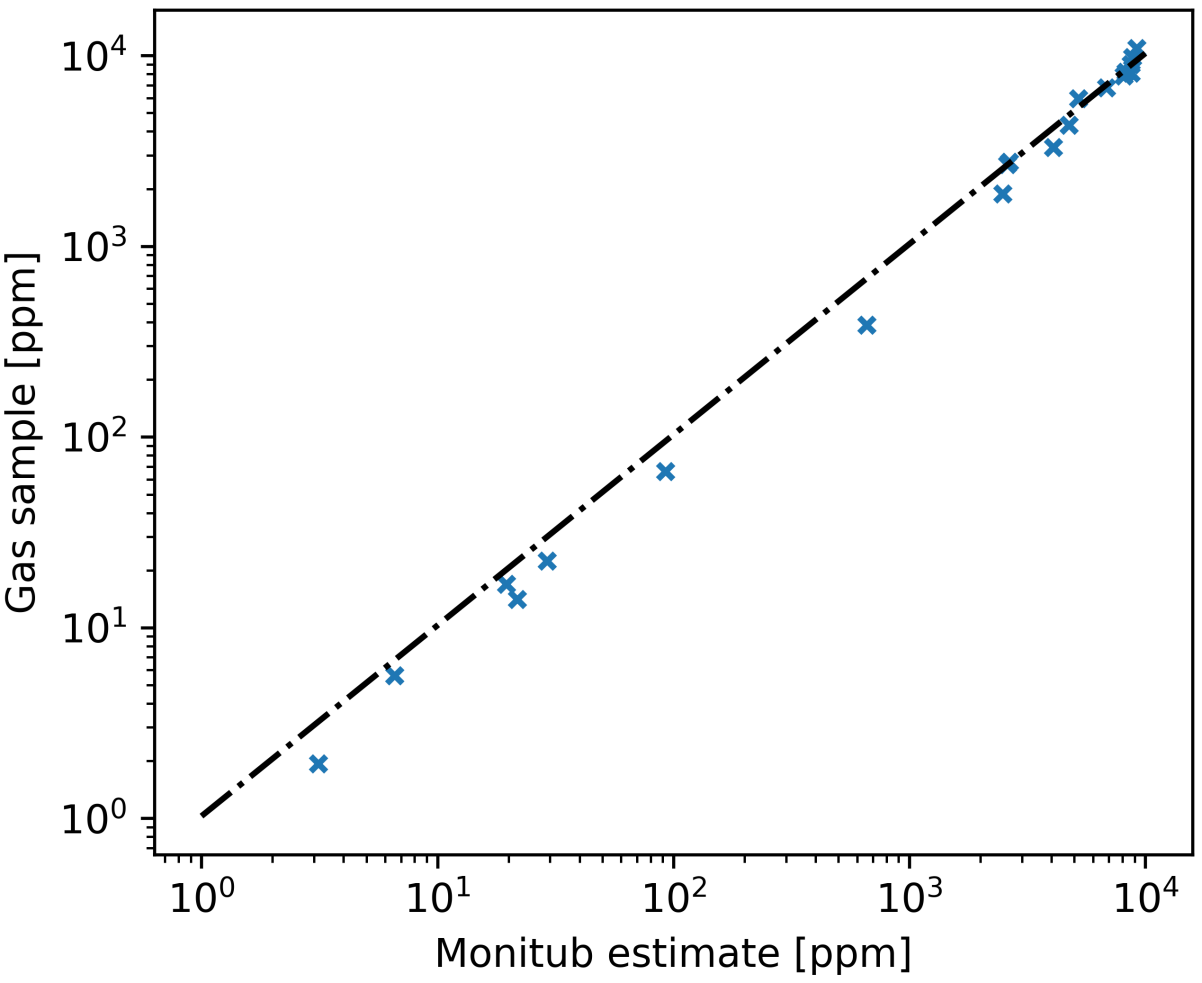
Comparison of recorded gas concentrations reported by Monitub and in situ gas samples taken during multiple deployments (blue cross). The dashed line represents a line of best fit with equation y=1.028x, $R^{2}=0.98$ and RMSE=535.47 ppm.

Additional headspace gas samples were taken in situ from 4 different SENS-0001 sensorboards deployed to Monitub AFCs, sampled from 22 to 170 days into the deployment. These samples were used to validate the calibration of the TGS2611-E00 sensor (Figure 10). The 20 samples showed high correlation (r>0.97) between the sampled ${\mathrm{CH}}_{4}$ concentration and the estimated concentration. This was persistent across a large range of concentrations (~2 ppm–~11,000 ppm), with a linear fit of slope 1.028. The fit is valid even at low concentrations (<2000 ppm, $R^{2}=0.82$ and RMSE=259.12 ppm), showing it is not being skewed by the higher concentrations.

### 3.3. Venting Sub-System Verification

**Figure 11:**
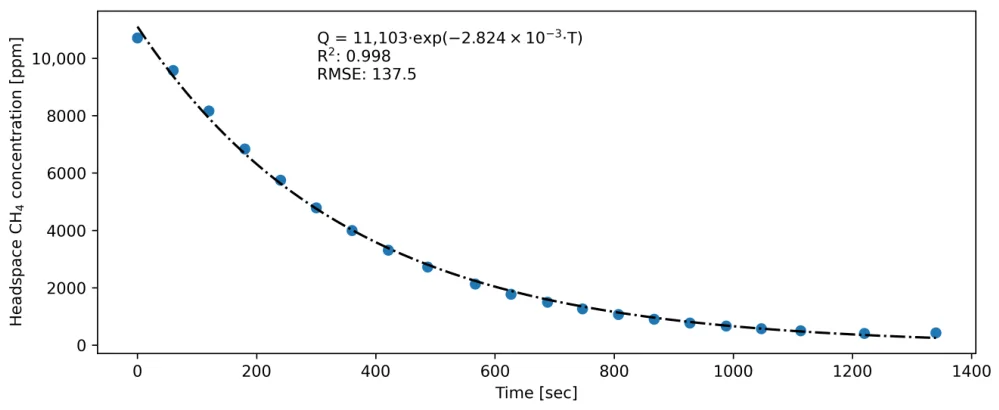
Experimental results of the venting sub-system, where measurements of headspace concentrations were taken once a minute (blue dots); an exponential function was fit to this experimental data (black dashed line).

Verification of the venting sub-system was conducted by introducing an initial concentration of ${\mathrm{CH}}_{4}$ into the headspace of a chamber. The venting sub-system was then manually activated whilst headspace concentrations were measured once a minute. Results showed that concentrations in the headspace were halved every 245 s of continual operation (Figure 11). With typical sampling intervals of 10 min, this would result in a reduction in headspace concentration to ~18% of pre-vented values. The venting operation equalises the air pressure inside the headspace of the chamber to the atmosphere (Appendix D), thus also reducing any systematic errors imposed by the heavier AFC.

### 3.4. Urban Lake Deployment

**Figure 12:**
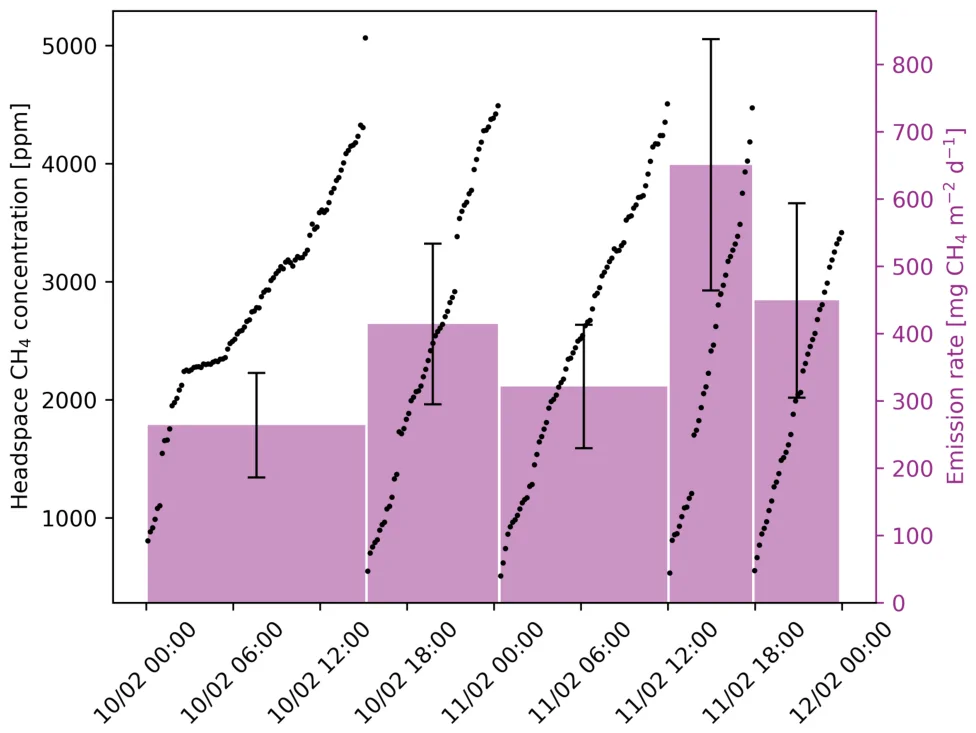
Data captured by the Monitub AFC in the wetlands section of the lake at The University of Queensland from 10 February 2025 to 12 February 2025. Black dots denote individual headspace measurements, with the purple bars denoting resultant flux estimates between vents, with associated error bars. Originally, the venting threshold was set to 5000 ppm, but due to older calibration constants this resulted in an effective threshold of ~4500 ppm.

From November 2024 to present (May 2026), a Monitub AFC has been deployed to the urban lake at The University of Queensland. Results from 10 to 11 February 2025 show headspace concentrations of the Monitub AFC increasing, with sharp jumps periodically indicating a likely ebullition event capture (Figure 12). This is important as this shows that the system is capable of distinguishing between ebullition and diffusion, allowing for direct attribution, something that is not possible in manual FCs. Once the headspace concentrations were over 4500 ppm, the chamber automatically vented. Between these vents, the units of the headspace concentration were converted (Appendix E.2) and an emission rate was calculated with associated error (Appendix E.3), showing a reasonably accurate result with an expected error of between 28.5% and 32.1%. It can also be observed that each vent cycle resulted in different patterns of the time series, with variance present in the time between vents, overall shape of the trace, and how many likely ebullition events are captured. This shows the importance of continual monitoring to capture the temporal variability of these emissions.

**Figure 13:**
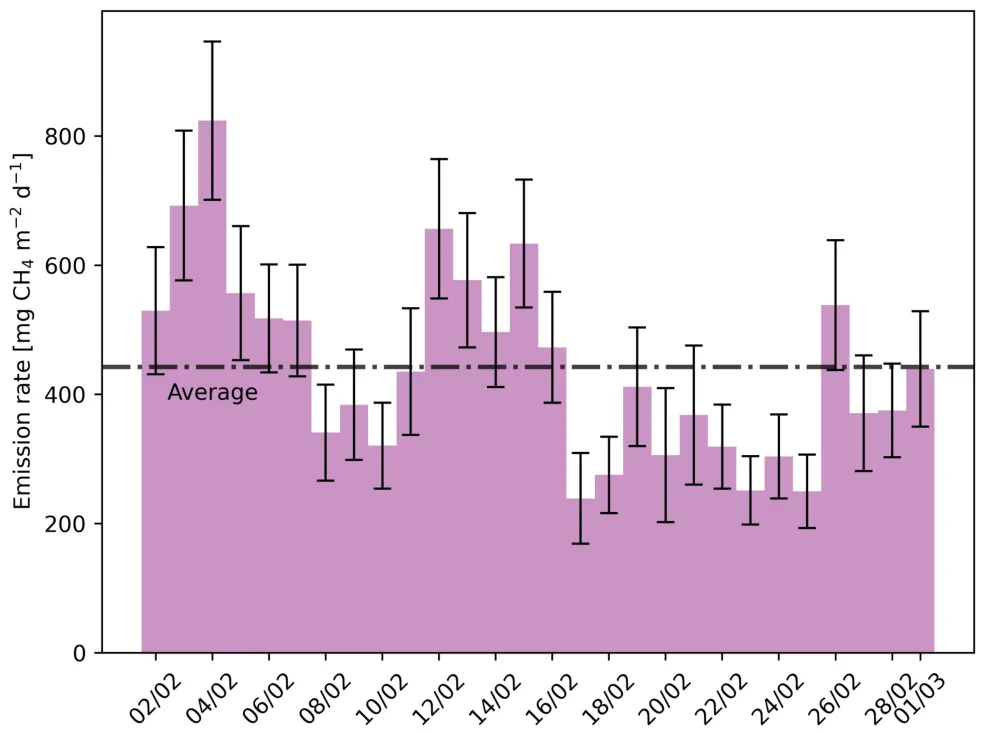
Daily emission rates (purple bars) captured by the Monitub AFC in the wetlands section of the lake at The University of Queensland from 2 February 2025 to 1 March 2025. Error bars were calculated using Equation (A31). The average rate is displayed as a black horizontal dashed line. A total of 86 venting cycles occurred during this period, averaging 3.1 per day.

Expanding the dataset to a month from 2 February 2025 to 2 March 2025, it can be seen that the Monitub AFC consistently captured ${\mathrm{CH}}_{4}$ emissions over a long time period (Figure 13). The rate and associated error were calculated as described in Appendix E.4. The results show differences in emission rates between days, with 164 of the 378 pairings of days (43.4%) having statistically distinct emission rates (Welch’s *t*-test, p<0.05), with 5 of the pairings being consecutive days. This is significant as it further shows the need for long-term monitoring of ${\mathrm{CH}}_{4}$ emissions, as single day measurements, such as conducted with manual FCs, would lead to vast under-/overestimation of average emission rates.

## 4. Discussion

The Monitub AFC has been shown to provide low-cost, real-time, and long-term monitoring of ${\mathrm{CH}}_{4}$ emissions from inland waterbodies. The system uses low-cost sensors, the TGS2611-E00 for ${\mathrm{CH}}_{4}$ (~$65 AUD), the SCD41 for ${\mathrm{CO}}_{2}$ (~$30 AUD), and the SHT41 for T/RH (~$5 AUD), to monitor the headspace of a mass-manufacturable FC (<$150 AUD). The TGS2611-E00 sensor has been used in other low-cost AFCs [36,42,48]; however, the calibration range has been significantly expanded, with a new upper range of 10,000 ppm (Table 1). This allows for monitoring in locations which are susceptible to ebullition, as this pathway is a direct release of ${\mathrm{CH}}_{4}$. The new calibration is also more generic, with only a single parameter being unique to each sensor, leading to less calibration data and, thus, fewer man-hours, being required for new sensors. This lower total cost allows for more systems to be made and deployed simultaneously, thus helping to constrain uncertainties surrounding the spatial heterogeneity of ${\mathrm{CH}}_{4}$ emissions.

Unlike manual FC designs, and some AFC designs (Table 1), the Monitub AFC is able to push data to an online dashboard, allowing for the remote monitoring of emissions. The dashboard also allows for the collation of multiple data streams, such as atmospheric conditions or other chambers, to provide an on-demand comparison of emission rates. Similar to other AFCs, the Monitub AFC is able to monitor headspace concentrations at a higher temporal rate than what can be achieved through manual FCs, allowing for the direct attribution of emissions pathways. An example of this was from the deployment to the urban lake, where likely ebullition events were visible as discontinuities in the time series, and each vent resulted in different emission patterns.

The deployment to the urban lake showed the long-term capabilities of the Monitub AFC, as it has been deployed since November 2024, with this deployment still continuing. This was achieved through its chamber design and venting sub-system. As the venting sub-system uses a pump and valve to perform its operation, the chamber is not lifted from the water, making it less likely for the chamber to be flipped by the wind and waves. This is enhanced by the chamber sitting 100 mm into the water, lowering the likelihood of waves unsealing the headspace and venting it unexpectedly. The venting sub-system maintains headspace concentrations within the sensor’s calibrated range, and removes errors imposed by equalisation with the water column, removing the need for field personnel to be present at the deployment location. The deployment also showed the necessity for long-term continual monitoring of these emissions, especially when ebullition is present, as changes in daily emissions were statistically significant. If a traditional FC was used, this could lead to under-/overestimation of emissions, supporting the findings in [21], with the minimum daily emission being 46% below the average and the maximum daily emission being 86% above the average. This monthly average is similar to that presented for the same waterbody in [23], being within the presented range for February (332 ± 312 mg $m^{-2}$ $d^{-1}$). The value measured by the Monitub AFC is believed to be higher than the average presented in [23], as from July 2022 to November 2023 the lake underwent a renewal project [64]. This included the introduction of a shallow wetlands section, which is where the Monitub was deployed, with the intent of the section being to improve water quality outflow to the main body, thus altering the organic matter regime in the new wetlands section.

**Figure A7:**
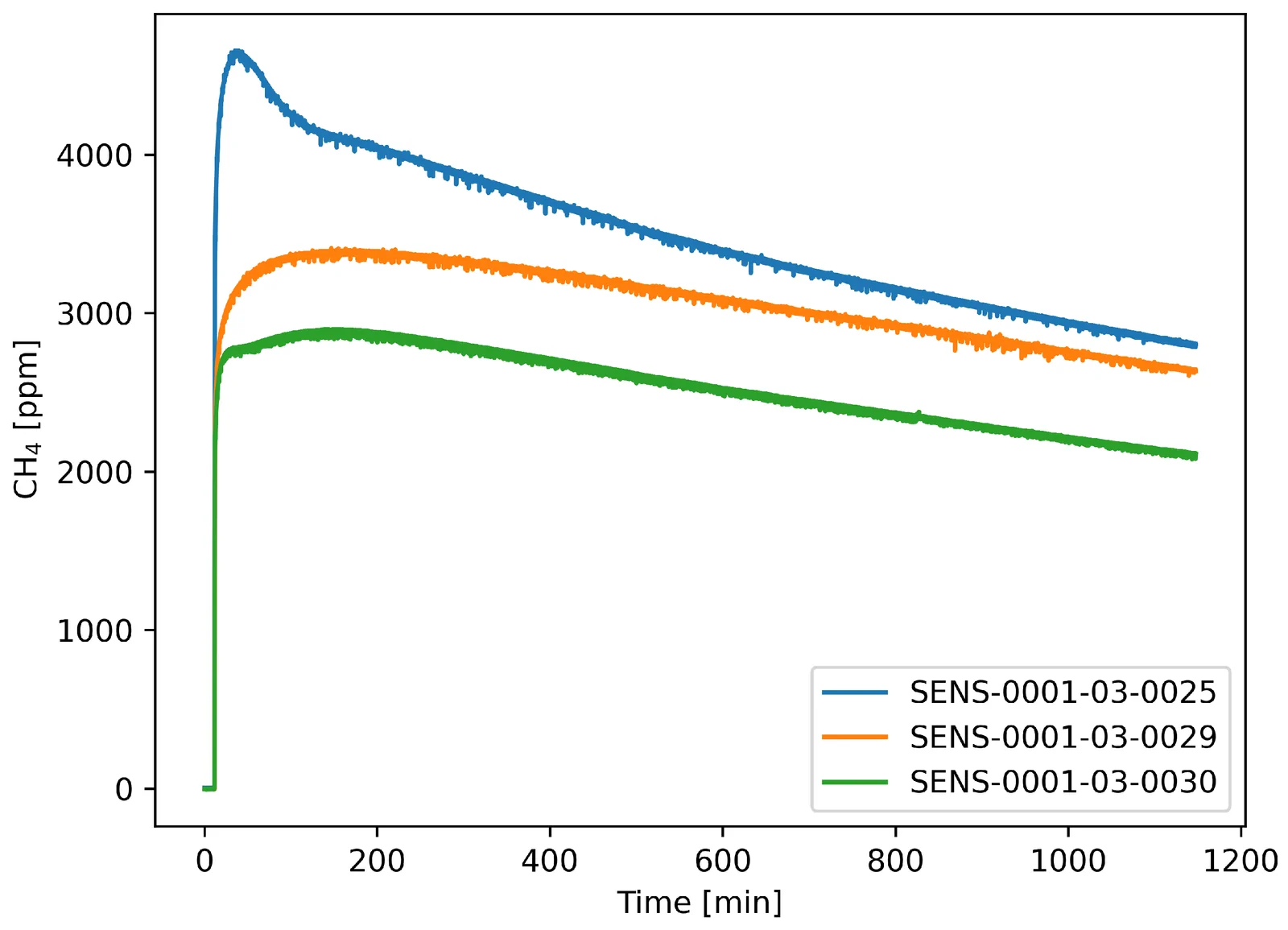
Post-validation results from two sensors (SENS-0001-03-0025 (blue) and SENS-0001-03-0030 (green)) which were deployed in a Monitub AFC on a reservoir for 218 days in areas with a high amount of ebullition activity. Post-validation was performed by sealing the sensors into a container with a reference sensor (SENS-0001-03-0029 (orange)) which has not been deployed and injecting an amount of gas standard. The container was left in a fume hood to slowly leak gas out through a tube. This shows a constant offset error after initial settling between the deployed sensors and the reference sensor of 180 ppm (SENS-0001-03-0025) and 520 ppm (SENS-0001-03-0030), representing a drift of 0.83 ppm $d^{-1}$ and 2.39 ppm $d^{-1}$.

**Figure A8:**
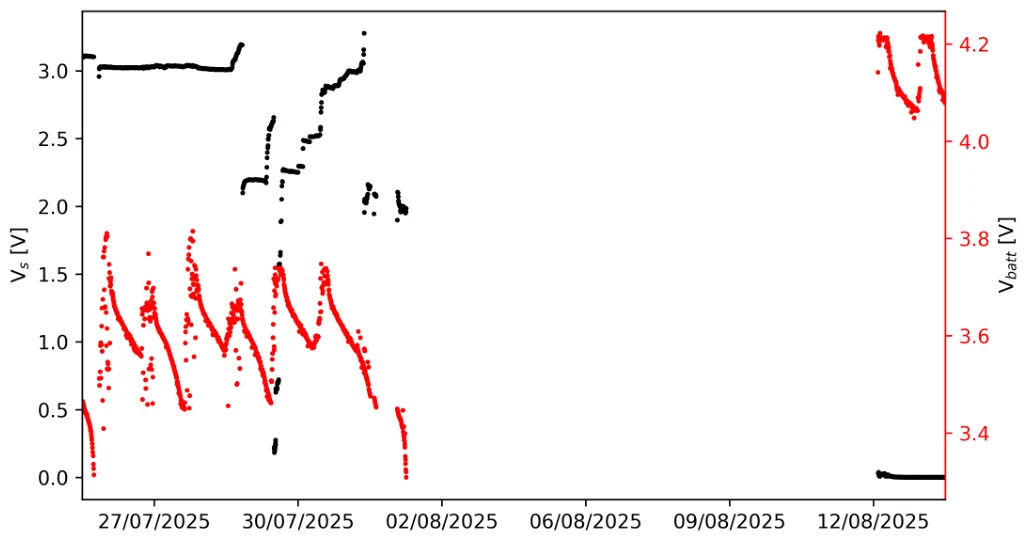
An example of the output of a sensor, the voltage across the load resistor (black dots), failing due to losing power to its heater, as represented by lowering battery voltage (red dots). This failure consisted of the sensor becoming an open circuit, causing a reading of 0 V across the load resistance, with normal atmospheric concentrations resulting in a voltage of 0.2–0.4 V. This shows the importance of having the heater always active whilst the sensor is in a sealed headspace.

**Figure A9:**
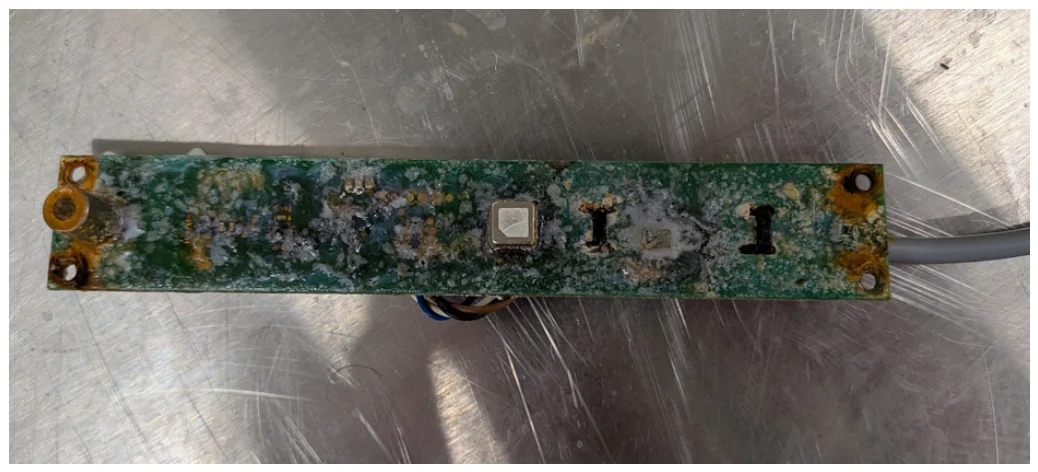
An example of condensation getting under the conformal coating of a SENS-0001 sensorboard, mainly affecting the supporting components of the PCB, causing failure of the sensorboard.

The conditions in the headspace of an AFC can be extreme, with internal humidities reaching higher than 100%RH, which can lead to condensation of water on sensors and supporting electronics, causing sensor drift and failure of components. To extend the usable life of the SENS-0001 sensorboard, an epoxy-based conformal coating was applied to the PCB, and care was taken as poor application of the coating can lead to areas not being sealed, leading to corrosion of the sensorboard (Figure A9). High humidity can also lead to damage to the TGS2611-E00 sensor itself, where condensation can form on the sensing element if the in-built heater is not powered at all times, such as if the datalogger’s battery dies and causes the sensor to lose power (Figure A8). Long-term deployments of the Monitub AFC have shown that if adequate steps are taken to protect the sensor there is limited drift, with the majority (85%) of validation samples presented in Figure 10 being taken 50–170 days into the deployment of the sensor, even with headspace humidities being predominantly above 100%RH for the length of the deployment. Post-deployment validation of some sensors also showed that deployment time can cause an offset error, modifying the value returned by the sensor (Figure A7); however, this offset is removed once emission rates are calculated, as it is a rate of change operation, thus lowering its influence.

## 5. Conclusions

Inland waterbodies are a major GHG source, with ${\mathrm{CH}}_{4}$ being a significant component of these emissions. The use of low-cost, real-time, long-term monitoring systems, such as Monitub, supports the monitoring of GHG emissions, as they provide understanding of the temporal and spatial patterns and variability of sources. This is especially true in inland waterbodies, where current statistical assumptions lead to high uncertainties regarding how much these systems contribute to global emissions [1]. The calibration presented for the low-cost TGS2611-E00 provides a wider range of calibration, up to 10,000 ppm, and a lower calibration requirement. However, further work on the calibration could bring measurement errors in line with those present in lower-range calibrations [36,48], thus leading to more reliable emissions results, as the sensor error is currently the largest contributor to emissions estimation errors. The design of Monitub allows for the long-term deployment and real-time monitoring of this sensor, thus providing the ability to capture more in situ measurements across both the temporal and spatial domains, helping to further inform total emissions estimates from these systems.

## Figures and Tables

**Table 1:** Comparison of the Monitub AFC to other AFCs in the literature.

Device	Calibration Maximum [ppm]	Reported Error/Fit	Chamber Volume [L]	Venting System	Dashboard
Monitub	10,000 ppm	535 ppm/0.98 (RMSE/$R^{2}$)	27.7	Active pumping of ambient air into headspace, with valve for outlet	Yes
AQUA-Flux [47]	2000 ppm ^1^	3.1 ppm (RMSE)	13.5	Chamber is tilted by linear actuator	No ^2^
Unnamed [38]	600 ppm	≥0.94 ($R^{2}$)	13.5	Undisclosed	No
AutoChamber [39]	100 ppm ^3^	0.92 ($R^{2}$)	27.0	Lifting lid design	No

^1^ This AFC had a separate automated bubble funnel for quantification of ebullitive fluxes. ^2^ Local communication is present. ^3^ Taken from maximum value presented in Figure 6a in [39].

**Figure A2:**
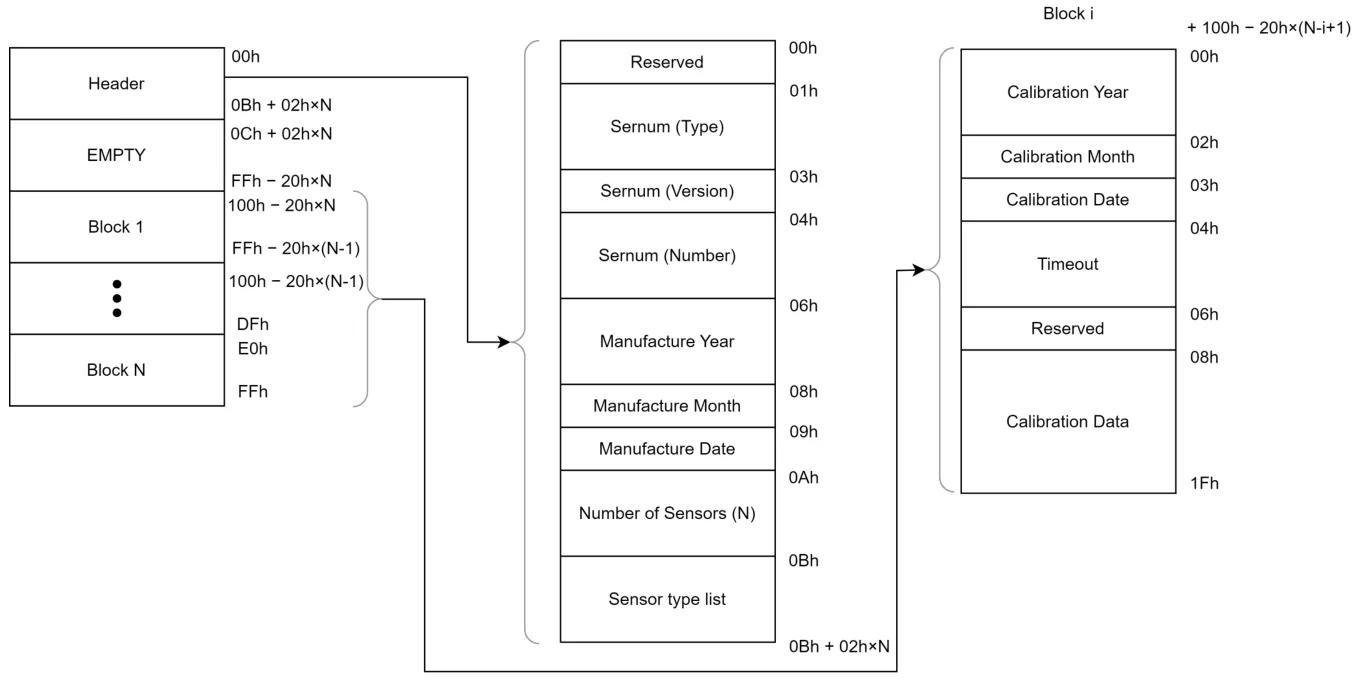
The generic memory layout of the MC24C02 EEPROM contained in the sensorboards. Containing a header (11 + 2*N* bytes), and a block of 32 bytes for each sensor on the board. The calibration data for each sensor is free-form and the datalogger treats it as a block of data, which is passed to the relevant sensor code. A maximum of 7 distinct sensors can be used for each sensorboard.

**Figure A4:**
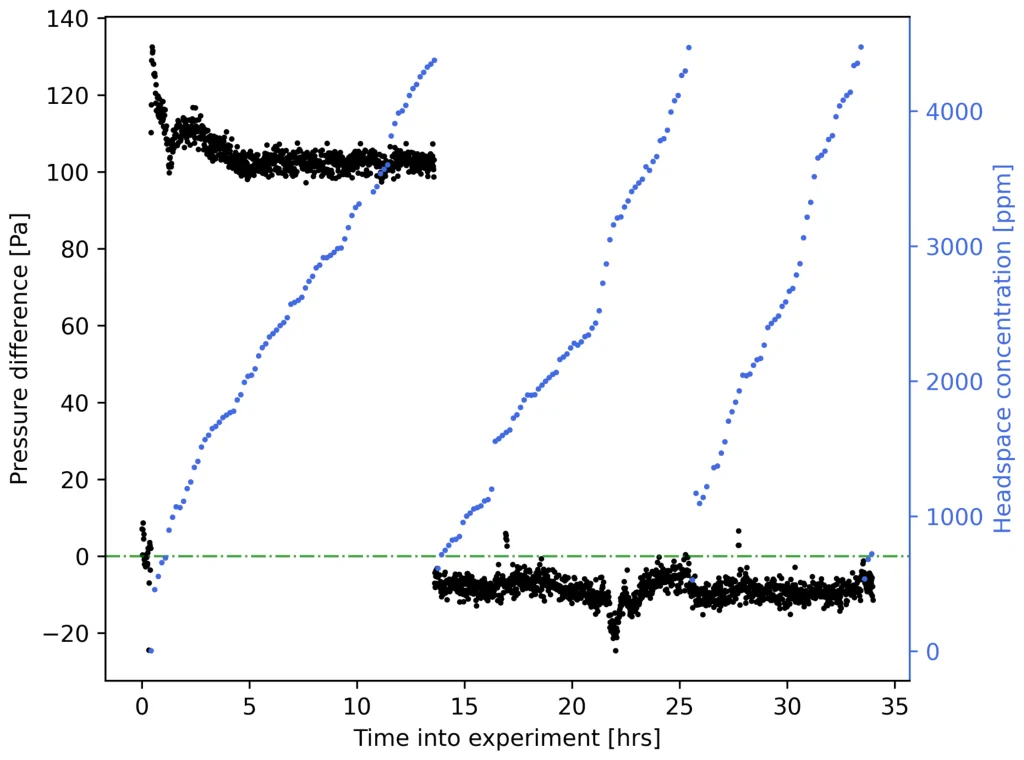
The difference in pressure between the headspace of an FC and the atmosphere (black dots), and the concentration of ${\mathrm{CH}}_{4}$ in the headspace (blue dots) over 35 h. The initial deployment forms a higher-pressure zone in the headspace due to the weight of the chamber. After the initial vent, this difference is removed due to equalisation with the atmosphere. For clarity equal pressure is designated by the green dashed line.

**Figure A5:**
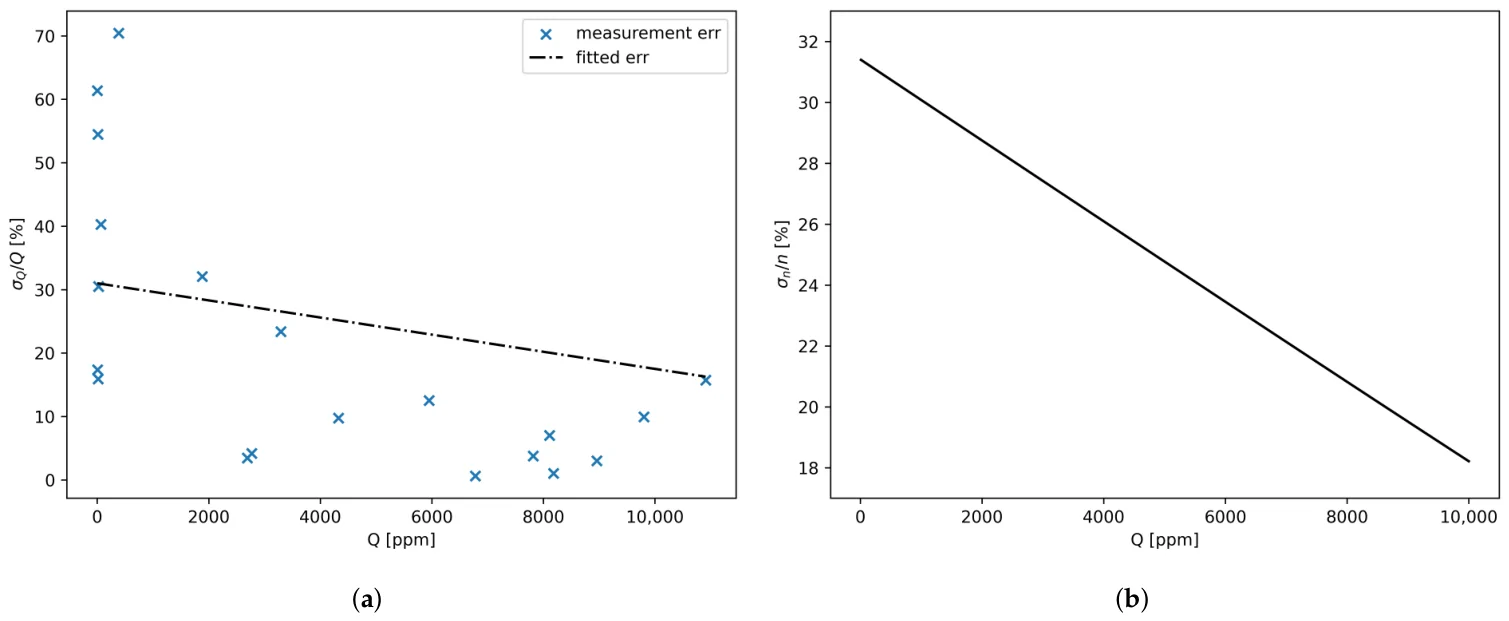
(**a**) Error associated with each of the headspace validation samples (blue x), with an approximate error line of $\sigma_{Q}/Q=0.31-1.35\cdot 10^{-5}Q$ (dashed line). (**b**) The calculated value of $\sigma_{n}/n$ at different concentrations of ${\mathrm{CH}}_{4}$ (*Q*).

**Figure A6:**
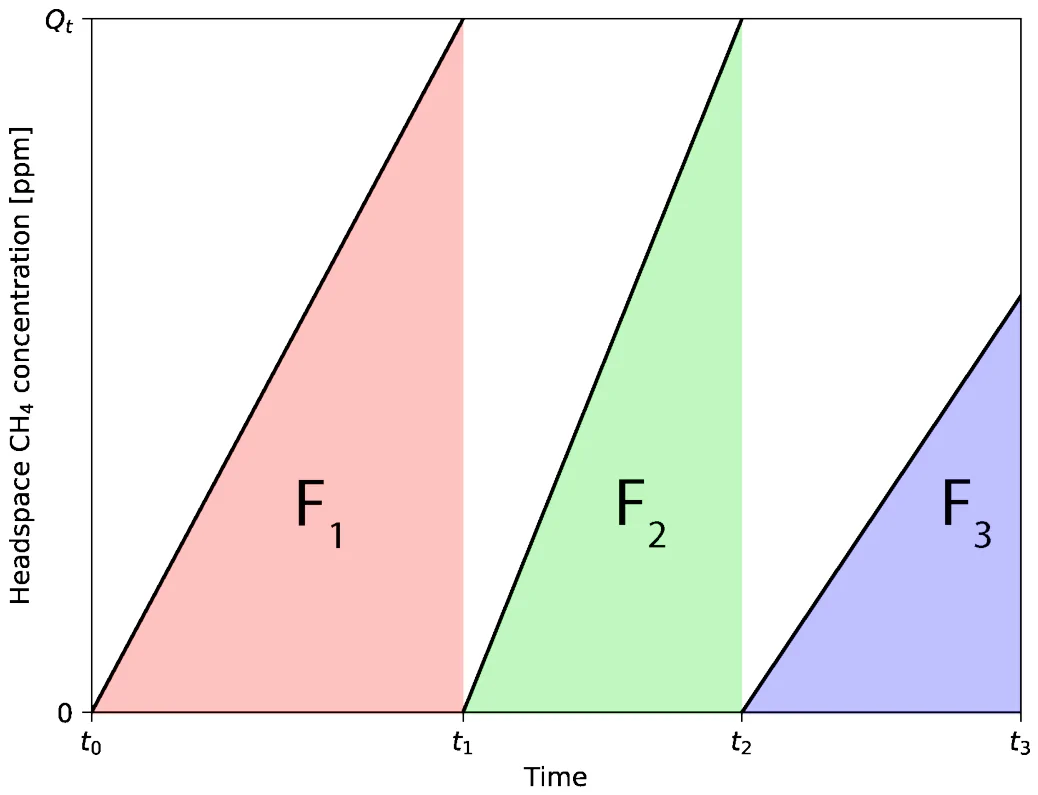
A conceptual view of what headspace concentrations within a Monitub might look like when multiple vents occur during a single day, or other such time period.

## Data Availability

Data is contained within the article or Supplementary Material.

## Supplementary Materials

The following supporting information can be downloaded at: https://www.mdpi.com/article/10.3390/s26165187/s1, raw data files for calibration, validation, and urban lake deployment.

## Appendix D. Chamber Pressure Differential Experiment

To investigate the pressure difference within a Monitub AFC, an experiment was conducted on The University of Queensland’s urban lake. This consisted of using two RBR solo pressure loggers, one on the shore next to the chamber to measure atmospheric pressure, and another within the headspace of the chamber. The pressure loggers were deployed to log simultaneously, with a short period where both were exposed to the atmosphere to determine any offsets between the sensors. The FC was then left for three venting cycles. The difference between the loggers (Figure A4) shows that initially a large (>100 Pa) pressure difference was experienced, due to the weight of the chamber. The difference was then significantly reduced once the first venting cycle occurred, with the differential stabilising at ~10 Pa, thus reducing this source of error in the sampling and conversion methods. The venting also settled the chamber to a depth of 100 mm into the water, which was stable between cycles, thus keeping the headspace volume relatively constant.

## Appendix E. Unit Conversions and Uncertainty Propagation

### Appendix E.1. Humidity Conversion

The SHT41 reports only temperature (*T*, °C) and relative humidity (RH, %); however, the calibration equation (Equation (1)) requires absolute humidity (*H*, kg $m^{-3}$). The conversion is performed by first finding the saturation vapour pressure ($P_{s}$, Pa) using the following equation and constants from [65]:

(A1) $$\begin{array}{cc} P_{s} & =P_{c}\exp\left[\frac{T_{c}}{T+273.15}\left(a_{1}\tau +a_{2}\tau^{1.5}+a_{3}\tau^{3}+a_{4}\tau^{3.5}+a_{5}\tau^{4}+a_{6}\tau^{7.5}\right)\right] \end{array}$$

(A2) $$\begin{array}{cc} \tau & =1-\frac{T+273.15}{T_{c}} \end{array}$$

The saturation vapour pressure is then scaled by relative humidity to get the actual vapour pressure ($P_{a}$, Pa):

(A3) $$P_{a}=P_{s}\times \frac{RH}{100}$$

Then, using the ideal gas law and molar mass of water (0.018015 kg $m^{-3}$), the value of *H* (kg $m^{-3}$) can be found:

(A4) $$H=\frac{P_{a}\times 0.018015}{R(T+273.15)}=\frac{P_{a}}{461.5(T+273.15)}$$

### Appendix E.2. Headspace Concentration Conversion

The calibration of the TGS2611-E00 sensor returns the headspace concentration in ppm; however, to use this for emission rate calculations it must be converted into mg, necessitating that it first be converted into moles. This was achieved using the ideal gas law:

(A5) pV=nRT

Using $p=p_{a}Q\cdot 10^{-6}$, where *Q* is the concentration, and $p_{a}$ is the atmospheric pressure in Pa, this transforms Equation (A5) into

(A6) $$n=\frac{p_{a}V_{h}\cdot 10^{-6}}{RT_{h}}Q$$

where $V_{h}$ and $T_{h}$ are the volume ($m^{3}$) and temperature (°K) of the headspace of the chamber. This is then converted to mg by multiplying by the molar mass of methane (16.04 g ${\mathrm{mol}}^{-1}$). The conversion uses atmospheric pressure instead of pressure within the headspace as this helps reduce sensor counts, and the venting sub-system allows for the equilisation of the pressure in the headspace of the chamber, and the atmosphere (Appendix D).

To propagate uncertainties through Equation (A6), we use $Q=Q_{0}\pm \sigma_{Q}$, $V_{h}=V_{h0}\pm \sigma_{V_{h}}$, $T_{h}=T_{h0}\pm \sigma_{T_{h}}$, and $p_{a}=p_{a0}\pm \sigma_{p_{a}}$, where $\sigma_{Q}$, $\sigma_{V_{h}}$, $\sigma_{T_{h}}$, and $\sigma_{p_{a}}$ are the absolute errors associated with each parameter. As Equation (A6) purely consists of multiplication and division of error-prone components, we can determine $\sigma_{n}$ (the uncertainty of moles) as follows:

(A7) $${\left(\frac{\sigma_{n}}{n}\right)}^{2}={\left(\frac{\sigma_{p_{a}}}{p_{a}}\right)}^{2}+{\left(\frac{\sigma_{V_{h}}}{V_{h}}\right)}^{2}+{\left(\frac{\sigma_{Q}}{Q}\right)}^{2}+{\left(\frac{\sigma_{T_{h}}}{T_{h}}\right)}^{2}$$

As previously found (Appendix D), the error related to using atmospheric pressure is minimal (~10 Pa), with absolute values being many orders of magnitude higher (~100,000 Pa); thus, we can see that this error component is insignificant (contribution of ~$10^{-8}$ to sum). Similarly, as $T_{h}$ is in kelvin and the reported sensor accuracy is 0.2 °C [58], this component can be safely ignored as well (contribution of <$10^{-6}$ to sum). Thus,

(A8) $${\left(\frac{\sigma_{n}}{n}\right)}^{2}\approx {\left(\frac{\sigma_{V_{h}}}{V_{h}}\right)}^{2}+{\left(\frac{\sigma_{Q}}{Q}\right)}^{2}$$

To determine error associated with $V_{h}$, let us assume that a chamber has been deployed with the headspace equalised to the atmosphere; thus, $p_{a0}=p_{a}$, $V_{0}=0.02768$ $m^{3}$ (volume of headspace when 100 mm deep in water), and $Q_{0}=2$ ppm of ${\mathrm{CH}}_{4}$. Now, assume the concentration overtime has increased to $Q_{1}$, $p_{a1}\approx p_{a0}=p_{a}$, and $T_{0}\approx T_{1}=T$. Letting $n_{0}$ and $n_{1}$ be the total moles within the headspace at the corresponding concentrations, then we have

(A9) $$\begin{array}{cc} n_{0} & =\frac{p_{a}V_{0}}{RT} \end{array}$$

(A10) $$\begin{array}{cc} n_{1} & =\frac{p_{a}V_{1}}{RT} \end{array}$$

(A11) $$\begin{array}{cc} n_{CH_{4}0} & =n_{0}Q_{0}\cdot 10^{-6} \end{array}$$

(A12) $$\begin{array}{cc} n_{CH_{4}1} & =n_{1}Q_{1}\cdot 10^{-6} \end{array}$$

Assuming that only ${\mathrm{CH}}_{4}$ has been added to the headspace, we have that $\Delta n=n_{1}-n_{0}=n_{CH_{4}1}-n_{CH_{4}0}$. Hence,

(A13) $$\begin{array}{cc} \Delta n=n_{1}-n_{0} & =\left(V_{1}-V_{0}\right)\frac{p_{a}}{RT} \end{array}$$

(A14) $$\begin{array}{cc} \wedge \;\Delta n=n_{CH_{4}1}-n_{CH_{4}0} & =\frac{p_{a}V_{1}}{RT}Q_{1}\cdot 10^{-6}-\frac{p_{a}V_{0}}{RT}Q_{0}\cdot 10^{-6} \end{array}$$

(A15) $$\begin{array}{cc} \Rightarrow (V_{1}-V_{0}) & =\left(V_{1}Q_{1}-V_{0}Q_{0}\right)\cdot 10^{-6} \end{array}$$

(A16) $$\begin{array}{cc} \; & =\left(\left(V_{0}+\left(V_{1}-V_{0}\right)\right)Q_{1}-V_{0}Q_{0}\right)\cdot 10^{-6} \end{array}$$

(A17) $$\begin{array}{cc} \; & =V_{0}\left(Q_{1}-Q_{0}\right)\cdot 10^{-6}+\left(V_{1}-V_{0}\right)Q_{1}\cdot 10^{-6} \end{array}$$

(A18) $$\begin{array}{cc} \Rightarrow \Delta V=(V_{1}-V_{0}) & =V_{0}\frac{\left(Q_{1}-Q_{0}\right)\cdot 10^{-6}}{1-Q_{1}\cdot 10^{-6}} \end{array}$$

When setting $Q_{0}=2$ and $Q_{1}=10,000$, we have that $\Delta V\approx 0.01V_{0}$. Hence, we see that a 1% difference in volume of the headspace is experienced purely by the additional ${\mathrm{CH}}_{4}$. However, as it was assumed that it was only ${\mathrm{CH}}_{4}$, the error used for the uncertainty propagation will be taken as 2% to account for other gases. Hence, Equation (A8) simplifies to

(A19) $${\left(\frac{\sigma_{n}}{n}\right)}^{2}\approx 4\cdot 10^{-4}+{\left(\frac{\sigma_{Q}}{Q}\right)}^{2}$$

To provide an estimate for $\sigma_{Q}/Q$, the error for each of the samples used for the sensors’ validation (Figure 10) was plotted (Figure A5a). A linear fit was added such that most of the error values were less than it, resulting in $\sigma_{Q}/Q\approx 0.31-1.35\cdot 10^{-5}Q$. This results in the final uncertainty approximation (Figure A5b) of

(A20) $${\left(\frac{\sigma_{n}}{n}\right)}^{2}\approx 4\cdot 10^{-4}+{\left(0.31-1.35\cdot 10^{-5}Q\right)}^{2}$$

### Appendix E.3. Emission Rate Calculations

To determine the emissions rate, a rate of change operation is performed between concentration readings. This results in an instantaneous rate of

(A21) $$F=\frac{n_{1}-n_{0}}{t_{1}-t_{0}}\cdot \frac{16.04}{A_{h}}$$

where $A_{h}$ is the area covered by the chamber ($m^{2}$), 16.04 is the molar mass of ${\mathrm{CH}}_{4}$ (g ${\mathrm{mol}}^{-1}$), and $t_{0}$ and $t_{1}$ are the times of the separate measurements (days). This results in *F* having the units g $m^{-2}$ $d^{-1}$. Replacing $\Delta n=n_{1}-n_{0}$ and $\Delta t=t_{1}-t_{0}$, we find the uncertainty as follows:

(A22) $${\left(\frac{\sigma_{F}}{F}\right)}^{2}={\left(\frac{\sigma_{\Delta n}}{\Delta n}\right)}^{2}+{\left(\frac{\sigma_{\Delta t}}{\Delta t}\right)}^{2}+{\left(\frac{\sigma_{A_{h}}}{A_{h}}\right)}^{2}$$

Assuming that $\sigma_{t_{1}}=\sigma_{t_{0}}=\sigma_{t}$, we find

(A23) $$\begin{array}{cc} {\left(\sigma_{\Delta n}\right)}^{2} & ={\left(\sigma_{n_{1}}\right)}^{2}+{\left(\sigma_{n_{0}}\right)}^{2} \end{array}$$

(A24) $$\begin{array}{cc} \Rightarrow {\left(\frac{\sigma_{\Delta n}}{\Delta n}\right)}^{2} & =\frac{{\left(\sigma_{n_{1}}\right)}^{2}+{\left(\sigma_{n_{0}}\right)}^{2}}{{\left(n_{1}-n_{0}\right)}^{2}} \end{array}$$

(A25) $$\begin{array}{cc} {\left(\sigma_{\Delta t}\right)}^{2} & =2{\left(\sigma_{t}\right)}^{2} \end{array}$$

(A26) $$\begin{array}{cc} \Rightarrow {\left(\frac{\sigma_{\Delta t}}{\Delta t}\right)}^{2} & =\frac{2{\left(\sigma_{t}\right)}^{2}}{{\left(t_{1}-t_{0}\right)}^{2}} \end{array}$$

As the timestamp of each sample is taken from an RTC and corrected by GPS, we can safely assume that $\sigma_{t}\leq 1$, and with sampling generally being every 10 min, we see that ${\left(\sigma_{\Delta t}/\Delta t\right)}^{2}<6\cdot 10^{-6}$, with this only decreasing with larger time difference between samples. Thus, Equation (A22) can be simplified to

(A27) $${\left(\frac{\sigma_{F}}{F}\right)}^{2}\approx \frac{{\left(\sigma_{n_{1}}\right)}^{2}+{\left(\sigma_{n_{0}}\right)}^{2}}{{\left(n_{1}-n_{0}\right)}^{2}}+{\left(\frac{\sigma_{A_{h}}}{A_{h}}\right)}^{2}$$

As $A_{h}$ is taken as the area that the chamber covers, it is independent of the current depth that the chamber sits in the water. However, as a single value is used between chambers, an error of 1% will be used to account for manufacturing differences. Hence,

(A28) $${\left(\frac{\sigma_{F}}{F}\right)}^{2}\approx \frac{{\left(\sigma_{n_{1}}\right)}^{2}+{\left(\sigma_{n_{0}}\right)}^{2}}{{\left(n_{1}-n_{0}\right)}^{2}}+10^{-4}$$

As seen with large time differences, larger sample differences cause this error to be significantly reduced. As an example, let us calculate a flux from when a theoretical chamber is deployed, or directly after a vent, to when the chamber next vents (at 5000 ppm). We can then approximate the error associated with this flux. For $Q_{0}=2$ ppm, $Q_{1}=5000$ ppm, $V_{h}=0.02768$ $m^{3}$, $T_{h}$ = 30 °C and $p_{a}=101,325$ Pa, we can use Equations (A6), (A20) and (A28) to find the following:

(A29) $$\frac{\sigma_{F}}{F}\approx 25\%$$

### Appendix E.4. Daily Emission Rate Conversion

As venting on the Monitub AFC is not necessarily time-dependent, as is the case when a headspace concentration threshold is used instead, it can result in multiple segmented sections within a day (Figure A6). To calculate a daily emission rate, a method to join the emission rates of each of these segments must be devised.

This is achieved through the weighted average of each segment’s rate, with the weight being time. Firstly, we calculate $F_{i}$ and $\sigma_{F_{i}}$ using Equations (A21) and (A28) from the first data point in the segment (directly after previous vent) to the last (the one that results in a vent). Next, we defined the weight as being $w_{i}=t_{i}-t_{i-1}$. We can then get a final flux, *F*, as

(A30) $$F=\frac{w_{1}}{w_{s}}F_{1}+\frac{w_{2}}{w_{s}}F_{2}+\frac{w_{3}}{w_{s}}F_{3}=\frac{1}{w_{s}}\sum w_{i}F_{i}$$

where $w_{s}=w_{1}+w_{2}+w_{3}=\sum w_{i}$. We can then also use these weights to provide an error associated with this sum:

(A31) $${\left(\sigma_{F}\right)}^{2}={\left(\frac{w_{1}}{w_{s}}\sigma_{F_{1}}\right)}^{2}+{\left(\frac{w_{2}}{w_{s}}\sigma_{F_{2}}\right)}^{2}+{\left(\frac{w_{3}}{w_{s}}\sigma_{F_{3}}\right)}^{2}=\frac{1}{{\left(w_{s}\right)}^{2}}\sum {\left(w_{i}\sigma_{F_{i}}\right)}^{2}$$

This method can be generalised to any number of segments within a day to allow for conversion to daily figures.
